# Systematic analysis of the IL‐17 receptor signalosome reveals a robust regulatory feedback loop

**DOI:** 10.15252/embj.2019104202

**Published:** 2020-07-21

**Authors:** Helena Draberova, Sarka Janusova, Daniela Knizkova, Tereza Semberova, Michaela Pribikova, Andrea Ujevic, Karel Harant, Sofija Knapkova, Matous Hrdinka, Viola Fanfani, Giovanni Stracquadanio, Ales Drobek, Klara Ruppova, Ondrej Stepanek, Peter Draber

**Affiliations:** ^1^ Laboratory of Immunity & Cell Communication BIOCEV First Faculty of Medicine Charles University Vestec Czech Republic; ^2^ Laboratory of Adaptive Immunity Institute of Molecular Genetics of the Czech Academy of Sciences Prague Czech Republic; ^3^ Laboratory of Mass Spectrometry BIOCEV Faculty of Science Charles University Prague Czech Republic; ^4^ Department of Haematooncology University Hospital Ostrava Ostrava Czech Republic; ^5^ Faculty of Medicine University of Ostrava Ostrava Czech Republic; ^6^ Institute of Quantitative Biology, Biochemistry, and Biotechnology SynthSys School of Biological Sciences University of Edinburgh Edinburgh UK

**Keywords:** IKKε, IL‐17, LUBAC, NEMO, TBK1, Immunology, Signal Transduction

## Abstract

IL‐17 mediates immune protection from fungi and bacteria, as well as it promotes autoimmune pathologies. However, the regulation of the signal transduction from the IL‐17 receptor (IL‐17R) remained elusive. We developed a novel mass spectrometry‐based approach to identify components of the IL‐17R complex followed by analysis of their roles using reverse genetics. Besides the identification of linear ubiquitin chain assembly complex (LUBAC) as an important signal transducing component of IL‐17R, we established that IL‐17 signaling is regulated by a robust negative feedback loop mediated by TBK1 and IKKε. These kinases terminate IL‐17 signaling by phosphorylating the adaptor ACT1 leading to the release of the essential ubiquitin ligase TRAF6 from the complex. NEMO recruits both kinases to the IL‐17R complex, documenting that NEMO has an unprecedented negative function in IL‐17 signaling, distinct from its role in NF‐κB activation. Our study provides a comprehensive view of the molecular events of the IL‐17 signal transduction and its regulation.

## Graphical abstract

Mass‐spectrometric investigation of the IL‐17 cytokine receptor signaling complex reveals a new role for the linear ubiquitin chain assembly complex (LUBAC) as a positive regulator of the IL‐17 pathway, and an inhibitory feedback loop involving NEMO‐recruited kinases TBK1 and IKKε.

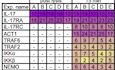

## Introduction

The interleukin 17 (IL‐17) is a major proinflammatory cytokine produced by Th17 cell lineage and several innate immune cell types (Harrington *et al*, 2005; Park *et al*, 2005; Cua & Tato, 2010). Studies of mouse models demonstrated that this cytokine is crucial for host defense against opportunistic fungal and bacterial species (Conti *et al*, 2009; Cho *et al*, 2010). In accord, patients impaired in IL‐17 signaling suffer from chronic mucocutaneous candidiasis (Puel *et al*, 2011; Conti & Gaffen, 2015). On the other hand, aberrant signaling via IL‐17 promotes pathogenesis of several autoimmune disorders, such as psoriasis, atopic dermatitis, rheumatoid arthritis, or multiple sclerosis (Brembilla *et al*, 2018) and therapeutic antibodies blocking IL‐17 or its receptor have been successfully used in clinic to treat severe plaque psoriasis (Bilal *et al*, 2018; Hawkes *et al*, 2018). Altogether, IL‐17 production and signal transduction must be subjected to a tight control to allow proper immune system response when required, yet preventing autoinflammatory diseases.

Interleukin‐17 receptor (IL‐17R) is composed of two widely expressed subunits IL‐17RA and IL‐17RC (Toy *et al*, 2006; Hu *et al*, 2010). Binding of dimeric IL‐17 leads to heterodimerization of the receptor (Ely *et al*, 2009; Liu *et al*, 2013; Goepfert *et al*, 2017) and recruitment of a cytoplasmic protein ACT1 (Chang *et al*, 2006; Qian *et al*, 2007). ACT1 was described to enhance the expression of genes encoding proinflammatory cytokines by stabilizing their mRNAs or by activating downstream signaling pathways leading to the activation of their transcription (Li *et al*, 2019). The gene‐activation pathways are dependent on the recruitment of E3 ubiquitin ligases from tumor necrosis factor (TNF) receptor‐associated factors (TRAFs) family, most prominently TRAF6. TRAF6 creates non‐degradative K63‐polyubiquitin linkages which serve as docking sites for a variety of signaling molecules and promote activation of downstream signaling pathways, especially mitogen‐activated protein kinase (MAPK) or nuclear factor‐κB (NF‐κB) and subsequent production of proinflammatory cytokines (Schwandner *et al*, 2000; Sonder *et al*, 2011). However, the mechanisms promoting and regulating IL‐17 signaling emanating directly from IL‐17 receptor are incompletely understood. The IL‐17‐induced activation of downstream pathways is surprisingly weak in comparison with other proinflammatory cytokines such as IL‐1α or TNF, although their receptors all employ the formation of non‐degradative polyubiquitin linkages and share multiple proximal signaling proteins (Kupka *et al*, 2016; Strickson *et al*, 2017; Li *et al*, 2019; McGeachy *et al*, 2019). The molecular basis for these differences is poorly defined. In addition to directly inducing activation of signaling pathways, IL‐17 can trigger stabilization of mRNA transcripts via ACT1 and TRAF2/5, which regulate mRNA stability either directly, or by modulating the activity of mRNA binding proteins ARID5A and HuR, splicing factor SF2, and endoribonuclease Regnase‐1 (Sun *et al*, 2011; Herjan *et al*, 2013, 2018; Somma *et al*, 2015; Amatya *et al*, 2018).

In this study, we established a novel methodical approach to analyze the assembly of the IL‐17 receptor signaling complex (IL‐17RSC) via mass spectrometry (MS), which revealed the composition of the complex and its stoichiometry, including a novel signaling mediator, linear ubiquitin chain assembly complex (LUBAC). Importantly, we uncovered a robust negative inhibitory loop mediated by NEMO‐recruited kinases TBK1 and IKKε that is specific for the IL‐17 pathway, explaining the enigmatic mechanism of a weak signaling response of cells to IL‐17 stimulation and showing a unique regulatory role of NEMO in the assembly of IL‐17RSC.

## Results

### Kinases TBK1 and IKKε are strongly and preferentially activated upon IL‐17 stimulation

**Figure EV1:**
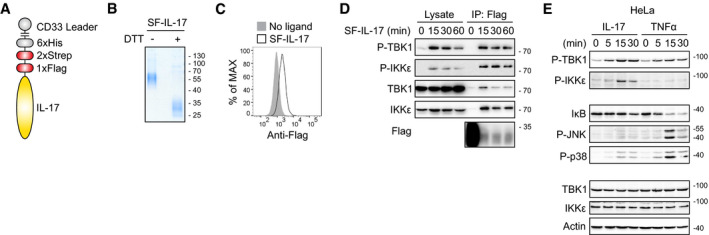
Isolation of IL‐17RSC reveals that TBK1 and IKKε kinases are strongly activated upon IL‐17 stimulation The schematic representation of recombinant Strep‐Flag-IL‐17 (SF‐IL-17) construct used in this study. Murine or human IL‐17 coding sequence lacking leader peptide was used.The purity and assembly of SF‐IL-17 was analyzed by SDS‐PAGE followed by Coomassie staining. Samples were either left untreated or reduced with dithiothreitol (DTT) to disrupt the covalent IL‐17 dimers.ST2 cells were incubated on ice in the presence or absence of SF‐IL-17. Subsequently, the cells were stained with fluorescently labeled anti‐Flag antibody and analyzed by FACS.ST2 cells were stimulated with SF‐IL-17 (500 ng/ml) for indicated time points or were left unstimulated and IL‐17 was added post‐lysis. Lysates were subjected to anti‐Flag immunoprecitation to isolate IL‐17RSC. The samples were analyzed by immunoblotting.HeLa cells were stimulated with IL‐17 (500 ng/ml) or TNF (500 ng/ml) for indicated time points and analyzed via immunoblotting. Source data are available online for this figure.

**Figure 1:**
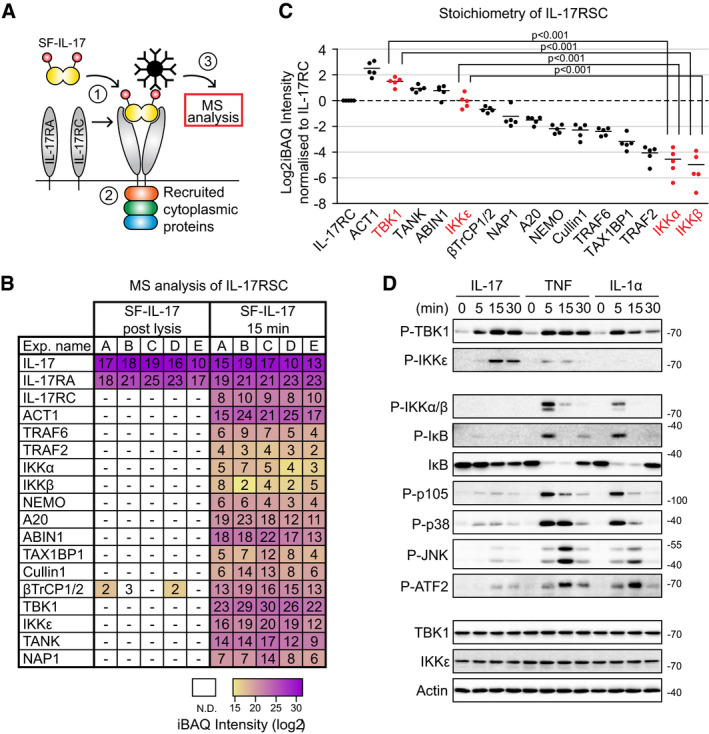
Kinases TBK1 and IKKε are major components of IL‐17RSC Schematic representation of IL‐17RSC isolation and analysis. Cells are stimulated with recombinant Strep‐Flag-IL‐17 (SF‐IL-17) (1), which leads to the crosslinking of its two receptor subunits and recruitment of cytoplasmic molecules (2). The whole complex is isolated upon cell lysis via tandem affinity purification of the ligand and analyzed by MS (3).ST2 cells were stimulated for 15 min with SF‐IL-17 (500 ng/ml), solubilized and IL‐17RSC was isolated via consecutive Flag and Strep immunoprecipitation and analyzed by MS. As a control, cells were first solubilized and SF-IL‐17 was added only post‐lysis. Number of identified peptides (unique + razor) and iBAQ intensities for each protein in five independent experiments are shown.The stoichiometry of IL‐17RSC calculated as the ratio between iBAQ intensities of individual IL‐17RSC components to iBAQ intensity of IL‐17RC. The recruitment of kinases TBK1 and IKKε as compared to related kinases IKKα and IKKβ is significantly enhanced. Mean from five performed MS experiments is shown, the statistical significance was determined using unpaired two‐tailed Student's *t*‐test.ST2 cells were stimulated with IL‐17 (500 ng/ml), TNF (50 ng/ml), or IL‐1α (50 ng/ml) for indicated time points and activation of signaling pathways was analyzed by immunoblotting. A representative of two independent experiments is shown. Source data are available online for this figure.

We aimed to resolve the composition of the IL‐17 receptor signaling complex formed upon the binding of IL‐17 to its receptors. For that purpose, we deployed a strategy for receptor‐complex analysis in which cells were stimulated with a recombinant dimeric IL‐17 (Fig EV1A–C), followed by the pull‐down of the whole signaling complex via the ligand's tandem affinity purification tag (2xStrep‐tag and 1xFlag‐tag) and MS analysis (Fig 1A). This approach offers the possibility to isolate only ligand‐engaged receptors forming membrane‐proximal signaling complexes via highly specific tandem affinity purification without the requirement for exogenous expression of tagged proteins in target cells. As a control, the ligand was added after the cell lysis, which did not induce assembly of the signaling complex. The IL‐17 stimulation might lead to post‐translational modifications of potential contaminants that would change their binding to the beads used for immunoprecipitation. In order to ensure that the identified proteins are bona fide components of the IL‐17RSC, we decided for relatively strict definition of background contaminants (as described in Table EV1).

The comparison between control and stimulated samples revealed a very specific and highly reproducible set of proteins recruited to IL‐17RSC. In contrast to IL‐17RA, we detected IL‐17RC only in stimulated but not control samples, which reflects that murine IL‐17RC binds IL‐17 only when it is associated with IL‐17RA (Kuestner *et al*, 2007). Importantly, we identified a number of previously known components of IL‐17RSC: core protein ACT1, non‐degradative ubiquitin ligases TRAF6 and TRAF2, deubiquitinase A20 and associated adaptors ABIN1 and TAX1BP1, a kinase complex NEMO/IKKα/IKKβ, and homologous kinases TBK1 and IKKε (Amatya *et al*, 2017). We also identified components of a degradative ubiquitin ligase complex consisting of βTrCP1/2 and Cullin1, which were previously reported to degrade ACT1 upon prolonged stimulation (Shi *et al*, 2011), although it was not known they are recruited directly to the IL‐17RSC. In addition, we identified proteins TANK and NAP1 that have not yet been connected to the IL‐17R pathway (Fig 1B and Table EV1). These two adaptors were reported to associate with TBK1 and IKKε (Chau *et al*, 2008; Helgason *et al*, 2013) and recruit them to the TNFR1 signaling complex (TNF‐RSC) (Lafont *et al*, 2018).

We subsequently calculated the stoichiometry between individual components of the complex using intensity‐based absolute quantification (iBAQ) (Schwanhausser *et al*, 2011). Murine IL‐17 binds first strongly to IL‐17RA and only subsequently can interact with IL‐17RC to form the complex in 1:2:1 stoichiometry between IL‐17RA:IL‐17:IL‐17RC (Ely *et al*, 2009; Liu *et al*, 2013; Goepfert *et al*, 2017). As IL‐17RC does not bind directly to IL‐17 in the post‐lysis control samples, we normalized the iBAQ values of individual proteins to IL‐17RC (Fig 1B). Surprisingly, TBK1 and IKKε were among the most abundant components of the complex, largely exceeding the related kinases IKKα and IKKβ that are crucial for NF‐κB activation (Fig 1C and Table EV1). We confirmed that both TBK1 and IKKε were recruited and phosphorylated on their activation Ser172 residue (Kishore *et al*, 2002; Ma *et al*, 2012) within the IL‐17RSC (Fig EV1D). High abundance of TBK1 and IKKε in the IL‐17RSC suggested that their activation might be a major signaling event triggered by IL‐17 stimulation. Indeed, IL‐17 strongly activated TBK1 and IKKε at a comparable or even higher level as the stimulation with strong proinflammatory stimuli TNF or IL‐1α (Fig 1D). In a sharp contrast, NF‐κB and MAPK signaling pathways were only weakly triggered by IL‐17. The same results were obtained in human cell line HeLa (Fig EV1E). Altogether, these data established that IL‐17 shows a unique preference for strong activation of TBK1 and IKKε kinases over other signaling events.

### Kinases TBK1 and IKKε negatively regulate IL‐17 signaling

Although our data showed that the activation of TBK1 and IKKε is likely the most prominent signaling event upon IL‐17 stimulation, the role of TBK1 and IKKε kinases in IL‐17 signaling is highly controversial. Ablation of IKKε or TBK1 alone was described to weakly inhibit MAPK signaling and IL‐17‐mediated stabilization of mRNA (Bulek *et al*, 2011; Herjan *et al*, 2018). In accord, stimulation of TBK1 and IKKε DKO cells with IL‐17 in the presence of TNF led to markedly decreased transcriptional response, indicating that both kinases are positive regulators of IL‐17 signaling responses (Tanaka *et al*, 2019). In striking contrast, overexpression of either TBK1 or IKKε in cells deficient for both these kinases led to inhibition of IL‐17‐induced downstream signaling (Qu *et al*, 2012), indicating that they are in fact negative regulators of IL‐17 signaling.

**Figure EV2:**
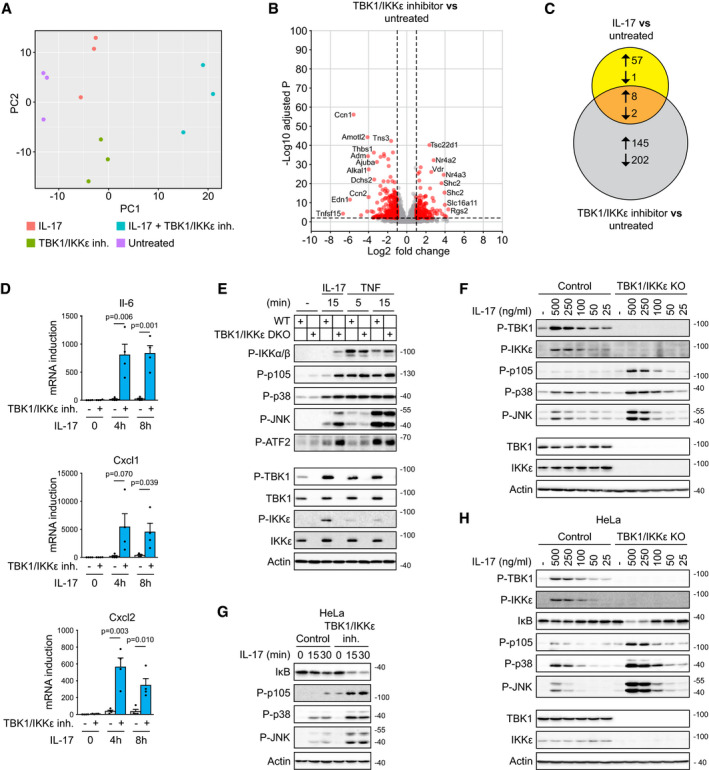
TBK1 and IKKε function as major inhibitors of IL‐17‐induced signaling and transcriptional response ST2 cells were incubated with or without TBK1/IKKε inhibitor MRT67307 (2 μM) for 30 min and subsequently were left untreated or stimulated for 2 h with IL‐17 (500 ng/ml). mRNA was isolated and subjected to RNA sequencing. The principal component analysis from three independent experiments is shown.Analysis of transcriptional response induced by treatment of ST2 cells with TBK1/IKKε inhibitor only. In red are transcripts considered to be significantly changed (log_2_ fold change > 1 or < −1, −log_10_ Benjamini–Hochberg adjusted *P*‐value > 2, based on analysis of three independent experiments). Names of several significantly upregulated transcripts are indicated.The Venn diagram representing the number of significantly changed transcripts upon treatment of cells with TBK1/IKKε inhibitor alone or with IL‐17 alone.ST2 cells pretreated or not with TBK1/IKKε inhibitor MRT67307 (2 μM) were left unstimulated or stimulated with IL‐17 (500 ng/ml) for 4 or 8 h, and induction of mRNA for selected genes was analyzed by real‐time PCR. Mean + SEM from four independent experiments is shown, and statistical significance was determined using unpaired two‐tailed Student's *t*‐test.ST2 wild types or TBK1/IKKε DKO cells were stimulated with IL‐17 (500 ng/ml) or TNF (50 ng/ml) for indicated time points and lysates were analyzed by immunoblotting.ST2 wild types or TBK1/IKKε DKO cells were stimulated with indicated concentration of IL‐17 for 15 min and lysates were analyzed by immunoblotting.HeLa cells pretreated or not with TBK1/IKKε inhibitor MRT67307 (2 μM) were stimulated with IL17 (500 ng/ml) for indicated time points and lysates were analyzed by immunoblotting.HeLla wild types or TBK1/IKKε DKO cells were stimulated with indicated concentration of IL‐17 for 15 min and lysates were analyzed by immunoblotting.Data information: Immunoblot results are representative of two (G, H) or three (F) independent experiments. Source data are available online for this figure.

**Figure 2:**
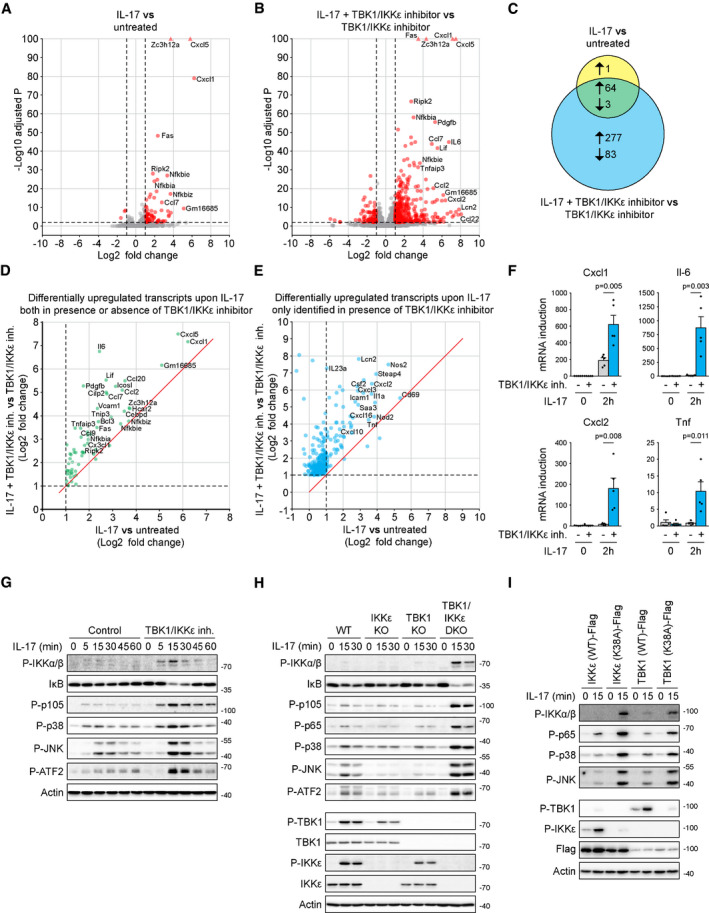
TBK1 and IKKε function in redundant manner as inhibitors of IL‐17‐induced signaling responses A, BST2 cells were either left untreated (A) or pretreated with TBK1/IKKε inhibitor MRT67307 (2 μM) for 30 min (B), followed by stimulation with IL‐17 (500 ng/ml) for 2 h. Transcription response induced by IL‐17 stimulation as compared to unstimulated cells was analyzed by RNA sequencing. In red are transcripts considered to be significantly changed (log_2_ fold change > 1 or < −1, −log_10_ Benjamini–Hochberg adjusted *P*‐value > 2, based on the analysis of three independent experiments; tringle is used for transcripts with −log_10_ adjusted *P*‐value > 100). Names of several significantly upregulated transcripts are indicated.CThe Venn diagram representing the number of significantly changed transcripts upon IL‐17 stimulation detected in (A) and (B).D, EComparison of IL‐17-induced transcriptional response in the presence or absence of TBK1/IKKε inhibitor. (D) Transcripts that are significantly changed in both conditions. (E) Transcripts that pass significance threshold for induction only in the presence of TBK1/IKKε inhibitor. Dashed lines indicate significantly upregulated transcripts (log_2_ fold change > 1); red lines separate transcripts that are more upregulated upon IL‐17 stimulation in the presence of TBK1/IKKε inhibitor as compared to IL‐17 alone.FST2 cells pretreated or not with TBK1/IKKε inhibitor MRT67307 (2 μM) were left unstimulated or stimulated with IL‐17 (500 ng/ml) for 2 h and induction of mRNA for selected genes was analyzed by real‐time PCR. Mean + SEM from five independent experiments is shown, and statistical significance was determined using unpaired two‐tailed Student's *t*‐test.GST2 cells were pretreated or not with TBK1/IKKε inhibitor MRT67307 (2 μM), stimulated with IL17 (500 ng/ml) for indicated time points and analyzed by immunoblotting.HST2 wild‐type, TBK1 KO, IKKε KO, or cells lacking both kinases (DKO) were stimulated with IL‐17 (500 ng/ml) as indicated and analyzed by immunoblotting.ITBK1/IKKε DKO cells reconstituted with either wild‐type or kinase dead mutant (K38A) version of both kinases were stimulated with IL‐17 (500 ng/ml) as indicated and analyzed by immunoblotting.Data information: Immunoblot results are representative of four (G) or three (H, I) independent experiments. Source data are available online for this figure.

In order to resolve the controversial issue concerning the role of these kinases in shaping IL‐17 responses, we employed RNA sequencing to analyze the transcription response following IL‐17 stimulation in the presence or absence of MRT67307, a highly specific inhibitor of both TBK1 and IKKε (Clark *et al*, 2011a) (Table EV2 and Fig EV2A). The comparison of unstimulated with IL‐17 stimulated cells showed upregulation of 65 genes, most of them being established targets of the IL‐17 signaling pathway (Fig 2A). Treatment of cells with MRT67307 alone induced subtle alternations of the transcriptome that were largely non‐overlapping with the effects of IL‐17 treatment (Fig EV2B and C). However, IL‐17‐induced pronounced changes in the transcriptional response of cells pretreated with MRT67307 (Fig 2B). Specifically, the inhibition of TBK1 and IKKε augmented the upregulation of almost all IL‐17 responsive genes (Fig 2C ans D). Moreover, multiple IL‐17 responsive genes (such as Tnf or Cxcl2) reached the significant level of upregulation only when the IL‐17 stimulation was performed in the presence of MRT67307 (Fig 2E). Real‐time PCR analysis confirmed that inhibition of TBK1 and IKKε markedly enhanced the IL‐17‐mediated upregulation of selected target genes after 2, 4, and 8 h of stimulation (Fig 2F and EV2D). Altogether, these data demonstrated that TBK1 and IKKε kinase activities lead to the general inhibition of IL‐17 transcriptional responses.

In the next step, we probed the role of TBK1 and IKKε in the IL‐17‐triggered proximal signaling pathways. The inhibition of TBK1 and IKKε activity dramatically enhanced the activation of NF‐κB and MAPKs (Fig 2G). Subsequently, we prepared cells deficient in TBK1, IKKε, or both using CRISPR/Cas9 approach. Ablation of either kinase alone led to weak suppression of responses to IL‐17 stimulation. In contrast, deficiency in both kinases led to strikingly enhanced activation of major signaling pathways, demonstrating absolute functional redundancy between TBK1 and IKKε in the inhibition of proximal IL‐17 signaling (Fig 2H). The reconstitution of TBK1/IKKε double knockout (DKO) cells with either wild‐type kinases TBK1 or IKKε, but not with their catalytically inactive versions, led to a strong inhibition of signaling (Fig 2I). As previously reported (Lafont *et al*, 2018), the ablation of both TBK1 and IKKε did not enhance signaling upon TNF, demonstrating that these two kinases have a unique role in the negative regulation of the IL‐17 signaling pathway (Fig EV2E). We observed strong TBK1/IKKε activation over a broad range of IL‐17 concentrations, which correlated with enhanced signaling in TBK1/IKKε DKO cells (Fig EV2F). Finally, we confirmed that concomitant deletion of TBK1 and IKKε or their chemical inhibition led to drastically enhanced IL‐17 signaling also in human HeLa cells (Fig EV2G and H). Altogether, our experimental evidence demonstrates that TBK1 and IKKε are strongly activated upon IL‐17 stimulation to provide potent inhibition of the downstream signaling in completely redundant manner.

### TBK1 and IKKε inhibit the recruitment of effector molecules into the IL‐17RSC

**Figure EV3:**
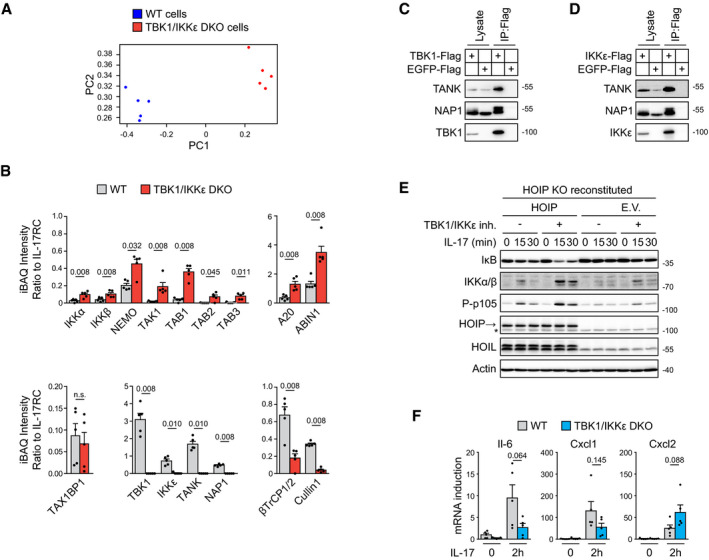
Ablation of TBK1 and IKKε leads to markedly changed composition of IL‐17RSC A, BST2 wild‐type or TBK1/IKKε DKO cells were stimulated for 15 min with SF‐IL-17 (500 ng/ml), solubilized and IL‐17RSC was isolated via consecutive Flag and Strep immunoprecipitation and analyzed by MS. (A) The principal component analysis of five independent experiments. (B) The ratio between iBAQ intensities of selected IL‐17RSC components to iBAQ intensity of IL‐17RC. Mean + SEM from five independent experiments is shown, and statistical significance was determined by two‐tailed Mann‐Whitney test.C, DST2 TBK1/IKKε DKO cells were reconstituted with Flag‐tagged TBK1 (C) IKKε (D) or GFP that serves as negative control. Cell lysates were subjected to anti‐Flag immunoprecipitation and analyzed via immunoblotting.EHOIP KO cells reconstituted with HOIP(WT) or empty vector were pretreated with TBK1/IKKε inhibitor MRT67307 (2 μM), stimulated with IL‐17 (500 ng/ml) as indicated and analyzed by immunoblotting. *indicates nonspecific band.FST2 wild types or TBK1/IKKε DKO were stimulated with IL‐17 (500 ng/ml) for 2 h and induction of mRNA for selected genes was analyzed by real‐time PCR. Mean + SEM from five independent experiments is shown, and statistical significance was determined using unpaired two‐tailed Student's *t*‐test.Data information: Immunoblot results are representative of two (C, D, E) independent experiments. Source data are available online for this figure.

**Figure 3:**
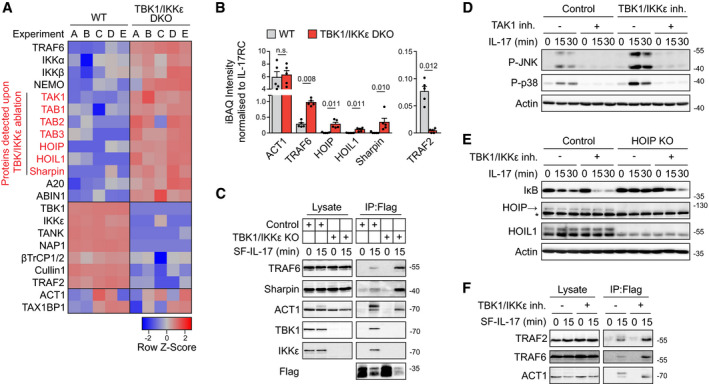
Kinases TBK1 and IKKε modulate the composition of IL‐17RSC A, BST2 wild‐type or TBK1/IKKε DKO cells were stimulated for 15 min with SF‐IL-17 (500 ng/ml) and solubilized and IL‐17RSC was isolated via consecutive Flag and Strep immunoprecipitation and analyzed by MS. (A) The heat map shows the row‐normalized *Z*‐Score calculated from log_2_ transformed iBAQ intensities from five independent experiments. (B) The ratio between iBAQ intensities of selected IL‐17RSC components to iBAQ intensity of IL‐17RC. Mean + SEM from five independent experiments is shown, and statistical significance was determined by two‐tailed Mann‐Whitney test.CST2 wild‐type or TBK1/IKKε DKO cells were stimulated with SF‐IL-17 (500 ng/ml) for 15 min or were left unstimulated and IL‐17 was added post‐lysis. Lysates were subjected to anti‐Flag immunoprecipitation to isolate IL‐17RSC and samples were analyzed by immunoblotting. ACT1 phosphorylation detected as upper band in WT cells is absent in TBK1/IKKε DKO cells.DCells were pretreated with TAK1 inhibitor 7‐oxozeanol (2 μM) and/or TBK1/IKKε inhibitor MRT67307 (2 μM), stimulated with IL‐17 (500 ng/ml) for indicated time points and lysates were analyzed by immunoblotting.EST2 wild‐type or HOIP‐deficient cells were pretreated with TBK1/IKKε inhibitor MRT67307 (2 μM), stimulated with IL‐17 (500 ng/ml) as indicated, solubilized, and analyzed by immunoblotting. *indicates unspecific band.FST2 cells pretreated or not with TBK1/IKKε inhibitor MRT67307 were stimulated with SF‐IL-17 for 15 min or were left unstimulated and IL‐17 was added post‐lysis. IL‐17RSC was isolated and analyzed via immunoblotting.Data information: Immunoblot results are representative of two (F), three (C, D) or four (E) independent experiments. Source data are available online for this figure.

To elucidate the molecular mechanism of how TBK1 and IKKε inhibit the IL‐17 signaling pathway, we compared the composition of IL‐17RSC in WT and TBK1/IKKε DKO cells via MS (Table EV3). The principal component analysis demonstrated that the absence of these two kinases led to markedly changed IL‐17RSC composition (Fig EV3A). We observed strong enrichment of non‐degradative ubiquitin ligases TRAF6 and linear ubiquitin chain assembly complex (LUBAC) consisting of HOIP, HOIL1, and Sharpin (Kirisako *et al*, 2006; Gerlach *et al*, 2011; Ikeda *et al*, 2011; Tokunaga *et al*, 2011) in TBK1/IKKε DKO cells (Fig 3A–C). The observation that LUBAC is reproducibly detectable only upon TBK1/IKKε ablation was intriguing, since LUBAC functions as a potent activator of NF‐κB in several proinflammatory complexes, such as TNF or IL‐1α (Hrdinka & Gyrd‐Hansen, 2017). Ubiquitin ligase activity of TRAF6 and LUBAC create K63‐ and M1‐ubiquitin linkages, respectively, which provide binding platforms for adaptors TAB 1/2/3 and associated kinase TAK1 and NEMO that recruits kinases IKKα and IKKβ (Kupka *et al*, 2016). In accord, the recruitment of all these proteins to IL‐17RSC was enhanced in TBK1/IKKε DKO cells (Figs 3A and EV3B). Interestingly, the recruitment of core adaptor ACT1 was not affected by the absence of both kinases, although ACT1 was not phosphorylated in TBK1/IKKε DKO cells (Fig 3A–C). On the other hand, proteins TANK and NAP1, two adaptors, constitutively associated with TBK1 and IKKε (Fig EV3C and D) were absent, while degradative ubiquitin ligase complex βTrCP1/2‐Cullin1 and TRAF2 recruitment was diminished in TBK1/IKKε DKO cells (Figs 3A and B, and EV3B).

Because LUBAC subunits and TAB/TAK1 complex were detected in IL‐17RSC upon ablation of TBK1/IKKε, we addressed their potential roles in the IL‐17 signaling. Inhibition of TAK1 blocked the IL‐17‐dependent activation of JNK and p38 both in the presence or absence of the TBK1/IKKε inhibitor, showing that TAK1 is indispensable for triggering the MAPK pathway in IL‐17 signaling (Fig 3D). Likewise, the LUBAC‐deficient HOIP KO cells showed substantially impaired activation of NF‐κB both in the presence and absence of the TBK1/IKKε inhibitor (Fig 3E) and reconstitution of HOIP KO cells with HOIP(WT), but not an empty vector, rescued IL‐17 signaling (Fig EV3E). These data show for the first time that LUBAC is a component of IL‐17RSC promoting the activation of NF‐κB. Overall, the data demonstrate that TBK1/IKKε‐mediated inhibition of IL‐17 proximal signaling is mediated by restriction of the recruitment of ubiquitin ligases TRAF6, LUBAC, and effector kinases TAK1 and IKKα/β to the IL‐17RSC.

While inhibition of TBK1/IKKε led to markedly enhanced activation of signaling and transcriptional responses (Figs 2 and EV2), cells deficient in TBK1/IKKε did promote gene‐activating signaling pathways, but were unable to induce accumulation of target cytokines mRNA (Fig EV3F). The most probable explanation for this apparent discrepancy is the differential recruitment of TRAF2 into the IL‐17RSC in these two scenarios. Whereas TRAF2 was depleted from the IL‐17RSC in the complete absence of TBK1/IKKε (Fig 3B), chemical inhibition of TBK1/IKKε kinase activity augmented the recruitment of TRAF2 to the complex (Fig 3F). The major role of TBK1/IKKε presence for TRAF2 recruitment in IL‐17RSC was intriguing. TRAF2 is not required for IL‐17‐induced proximal signaling; however, it promotes stabilization of target transcripts via several proteins regulating mRNA stability (Sun *et al*, 2011; Herjan *et al*, 2013, 2018; Somma *et al*, 2015; Amatya *et al*, 2018).

Altogether, our data show that TBK1/IKKε kinases have a dual role in IL‐17RSC function: (i) Enzymatic activity of TBK1/IKKε leads to the inhibition of TRAF6 recruitment and markedly decreased MAPK and NF‐κB signaling, while (ii) kinase activity‐independent adaptor role of TBK1/IKKε promotes recruitment of TRAF2, a regulator of mRNA stability of proinflammatory cytokines (Swaidani *et al*, 2019).

### TBK1 and IKKε are recruited to TRAF6‐generated ubiquitin linkages via NEMO

**Figure 4:**
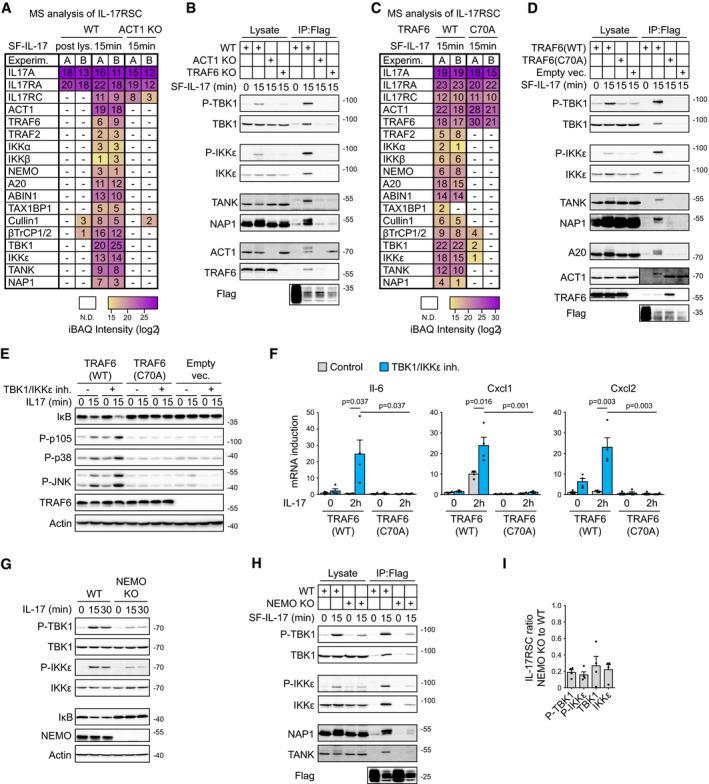
Enzymatic activity of TRAF6 and NEMO is crucial for the recruitment of both TBK1 and IKKε to IL‐17RSC AST2 wild‐type and ACT1‐deficient cells were stimulated for 15 min with SF‐IL-17 (500 ng/ml) and solubilized and IL‐17RSC was isolated via consecutive Flag and Strep immunoprecipitation and analyzed by MS. As a control, cells were first solubilized and SF-IL‐17 was added only post‐lysis. Number of identified peptides (unique + razor) and iBAQ intensities for each protein in two independent experiments are shown.BST2 wild‐type, ACT1 KO or TRAF6 KO cells were stimulated with SF‐IL-17 (500 ng/ml) as indicated or were left unstimulated and IL‐17 was added post‐lysis. Lysates were subjected to anti‐Flag immunoprecipitation to isolate IL‐17RSC and samples were analyzed by immunoblotting.CST2 cells deficient in TRAF6 reconstituted with TRAF6(WT) or enzymatically inactive TRAF6(C70A) were stimulated for 15 min with SF‐IL-17 (500 ng/ml), solubilized and IL‐17RSC was isolated via consecutive Flag and Strep immunoprecipitation and analyzed by MS. Number of identified peptides (unique + razor) and iBAQ intensities for each protein in two independent experiments are shown.DTRAF6‐deficient cells reconstituted with TRAF6(WT), TRAF6(C70A), or empty vector were stimulated with SF‐IL-17 (500 ng/ml) as indicated or were left unstimulated and IL‐17 was added post‐lysis. IL‐17RSC was isolated from lysates by anti‐Flag immunoprecipitation and analyzed by immunoblotting. Short and long exposure of the same membrane stained for Act1 are shown for lysate and IP samples, respectively.E, FTRAF6‐deficient cells reconstituted with the indicated constructs were pretreated or not with TBK1/IKKε inhibitor MRT67307 (2 μM) and stimulated with IL‐17 (500 ng/ml) for indicated time. (E) The activation of signaling pathways analyzed by immunoblotting. (F) The induction of mRNA for selected genes analyzed by real‐time PCR. Mean + SEM from four independent experiments is shown, and statistical significance was determined using unpaired two‐tailed Student's *t*‐test.GST2 cells wild‐type or NEMO KO were stimulated with IL‐17 (500 ng/ml) for indicated time points and solubilized and analyzed by immunoblotting.H, IST2 wild‐type or NEMO KO cells were stimulated with SF‐IL-17 (500 ng/ml) as indicated or were left unstimulated and IL‐17 was added post‐lysis. Lysates were subjected to anti‐Flag immunoprecipitation to isolate IL‐17RSC. Samples were analyzed by immunoblotting (H). In addition, results from four independent experiments were quantified by densitometry and mean + SEM from four independent experiments is shown (I).Data information: Immunoblot results are representative of two (B, E), three (D), or four (G–I) independent experiments. Source data are available online for this figure.

**Figure EV4:**
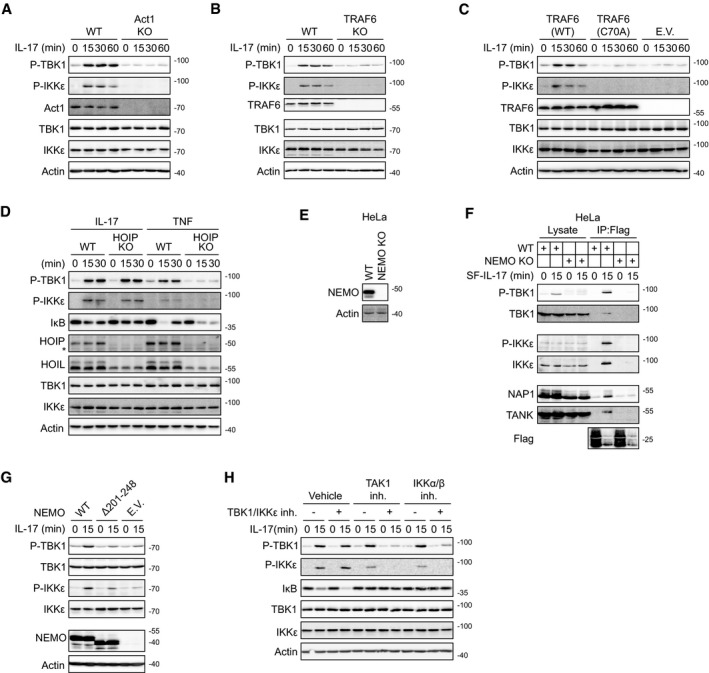
IL‐17‐induced TBK1 and IKKε activation requires TRAF6 and NEMO, but not HOIP A, BST2 cells wild‐type, ACT1 KO (A), or TRAF6 KO (B) were stimulated for indicated time points with IL‐17 (500 ng/ml) and lysates were analyzed by immunoblotting.CST2 cells deficient in TRAF6 were reconstituted with TRAF6(WT), enzymatically inactive TRAF6(C70), or empty vector and stimulated with IL‐17 (500 ng/ml) for indicated time points. Lysates were analyzed by immunoblotting.DST2 cells wild‐type or HOIP KO were stimulated with IL‐17 (500 ng/ml) or TNF (50 ng/ml) as indicated and lysates were analyzed by immunoblotting. *indicates unspecific band.ENEMO‐deficient HeLa cells were analyzed by immunoblotting.FHeLa wild‐type or NEMO KO cells were stimulated with SF‐IL-17 for 15 min or were left unstimulated and IL‐17 was added post‐lysis. Lysates were subjected to anti‐Flag immunoprecipitation to isolate IL‐17RSC, and samples were analyzed by immunoblotting.GNEMO‐deficient ST2 cells were reconstituted with NEMO(WT), NEMO (Δ201–248) or empty vector and stimulated with IL‐17 (500 ng/ml) as indicated. Activation of signaling pathways was analyzed upon cell lysis by immunoblotting.HST2 cells were pretreated with either IKKα/IKKβ inhibitor TPCA1 (10 μM), TAK1 inhibitor 7‐oxozeanol (2 μM), TBK1/IKKε inhibitor MRT67307 (2 μM), or their combination as indicated, stimulated or not with IL‐17 (500 ng/ml) for 15 min and analyzed by immunoblotting.Data information: Experiments are representative of two (A, C, D, H) or three (B, F, G) independent experiments. Source data are available online for this figure.

To elucidate the molecular mechanism underlying the TBK1 and IKKε function, we addressed how these kinases are recruited into the IL‐17RSC. First, we observed that cells deficient in ACT1, the very proximal IL‐17R‐interacting protein (Chang *et al*, 2006; Qian *et al*, 2007), show completely disrupted IL‐17RSC formation (Fig 4A and Table EV4) and disabled the activation of TBK1 and IKKε upon IL‐17 stimulation (Fig EV4A). The main role of ACT1 is to recruit and activate a non‐degradative E3 ubiquitin ligase TRAF6 (Schwandner *et al*, 2000). Accordingly, the presence of both ACT1 and TRAF6 is crucial for the recruitment of TBK1, IKKε, and associated adaptors TANK and NAP1 to IL‐17RSC and activation of both kinases (Figs 4B and EV4B). To elucidate whether TRAF6 enzymatic activity is necessary for the recruitment of these molecules, we reconstituted TRAF6 KO cells with either wild‐type TRAF6(WT) or enzymatically inactive TRAF6(C70A) mutant and analyzed the composition of IL‐17RSC via MS. The formation of the IL‐17RSC was largely disrupted in cells expressing the enzymatically inactive TRAF6(C70A) including the recruitment and activation of TBK1 and IKKε. We also noted that TRAF6 enzymatic activity was necessary for the recruitment of the inhibitory deubiquitinase A20 (Figs 4C and D, and EV4C and Table EV4). A20 was previously shown to modulate IL‐17 signaling, but was proposed to bind directly to IL‐17RA (Garg *et al*, 2013). However, our data imply that A20 complex is recruited via non‐degradative polyubiquitin linkages. Interestingly, TRAF6(C70A) recruitment to IL‐17RSC was markedly enhanced in comparison with TRAF6(WT) (Fig 4D), further demonstrating that IL‐17RSC assembly is regulated by potent negative feedback loop that is triggered downstream of TRAF6 activation.

The recruitment of TBK1 and IKKε to TNF‐RSC requires linear ubiquitin linkages synthetized by LUBAC (Lafont *et al*, 2018; Xu *et al*, 2018). In contrast, our data show that LUBAC is only very weakly recruited to IL‐17RSC and the knockout of HOIP, the main enzymatic subunit of LUBAC, did not prevent the activation of TBK1 and IKKε via IL‐17 (Fig EV4D). These data show that TRAF6, but not LUBAC, activity is indispensable for TBK1 and IKKε activation via IL‐17. Indeed, cells lacking the TRAF6 enzymatic activity are completely unresponsive to IL‐17 even in the presence of TBK1 and IKKε inhibitor (Fig 4E and F).

NEMO is crucial for the recruitment of IKKα and IKKβ to a variety of signaling complexes. However, it was also shown to recruit TANK and NAP1 to the M1‐ubiquitin chains in the TNF‐RSC (Chariot *et al*, 2002; Lafont *et al*, 2018). Since NEMO binds to TRAF6‐mediated K63‐ubiquitin as well (Ea *et al*, 2006; Wu *et al*, 2006; Laplantine *et al*, 2009), we hypothesized that NEMO recruits TANK and NAP1 adaptors bound to TBK1 and IKKε to the IL‐17RSC. Indeed, NEMO‐deficient cells failed to efficiently activate TBK1 and IKKε upon IL‐17 stimulation (Fig 4G). Accordingly, the recruitment of these kinases and their activation within IL‐17RSC was reduced by approximately 80% in the absence of NEMO in both murine and human cell lines as revealed by the quantification of Western blots (Figs 4H and I, and EV4E and F). Activation of TBK1/IKKε upon IL‐17 signaling was rescued in NEMO KO cells reconstituted with NEMO(WT), but not NEMO(Δ201–248) lacking TANK/NAP1‐binding domains (Chariot *et al*, 2002; Lafont *et al*, 2018) or an empty vector (Fig EV4G). Altogether, NEMO is the major protein connecting TBK1 and IKKε with K63‐linkages formed by TRAF6.

TBK1 and IKKε activation requires phosphorylation of Ser172 located in the kinase domain activation loop, which can be mediated either by TBK1/IKKε themselves or by IKKα/β (Ma *et al*, 2012; Larabi *et al*, 2013). In accord, only combined inhibition of IKKα/β and TBK1/IKKε kinases prevented TBK1/IKKε phosphorylation, while inhibition of IKKα/β alone or their upstream kinase TAK1 had no apparent effect (Fig EV4H). These results provide further evidence that NEMO‐associated kinases IKKα/β might contribute to, but are not essential for, TBK1/IKKε activation.

### NEMO has unprecedented dual role in the IL‐17 signaling

**Figure EV5:**
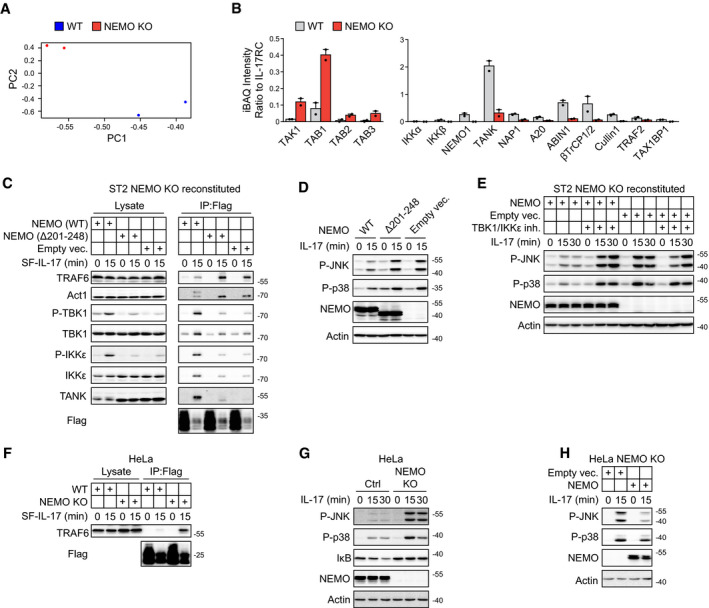
NEMO mediates recruitment of TBK1 and IKKε to inhibit IL‐17RSC complex assembly and downstream signaling A, BST2 wild‐type or NEMO KO cells were stimulated for 15 min with SF‐IL-17 (500 ng/ml), solubilized and IL‐17RSC was isolated via consecutive Flag and Strep immunoprecipitation and analyzed by MS. (A) The principal component analysis of two independent experiments. (B) The ratio between iBAQ intensities of selected IL‐17RSC components to iBAQ intensity of IL‐17RC. Mean + SEM from two independent experiments is shown.C, DNEMO‐deficient ST2 cells were reconstituted with NEMO(WT), NEMO (Δ201–248) lacking TANK/NAP1-binding site or empty vector and stimulated with SF‐IL-17 (500 ng/ml) for 15 min or were left unstimulated and IL‐17 was added post‐lysis. Lysates were subjected to anti‐Flag immunoprecipitation to isolate IL‐17RSC (C) or tested for the activation of signaling pathways (D) and samples were analyzed by immunoblotting.ENEMO‐deficient ST2 cells were reconstituted with NEMO(WT) or empty vector, treated or not with TBK1/IKKε inhibitor MRT67307 (2 μM) and stimulated with SF‐IL-17 (500 ng/ml) as indicated. Activation of signaling pathways was analyzed upon cell lysis by immunoblotting.F, GHeLa wild‐type or NEMO KO cells were stimulated with SF‐IL-17 (500 ng/ml) for 15 min or were left unstimulated and IL‐17 was added post‐lysis. Lysates were subjected to anti‐Flag immunoprecipitation to isolate IL‐17RSC (F) or tested for the activation of signaling pathways (G) and samples were analyzed by immunoblotting.HNEMO‐deficient HeLa cells were reconstituted with NEMO(WT) or empty vector and stimulated with IL‐17 (500 ng/ml) as indicated. Cells were solubilized and analyzed by immunoblotting.Data information: Immunoblot results are representative of two (C, D, E, G, H) or four (F) independent experiments. Source data are available online for this figure.

**Figure 5:**
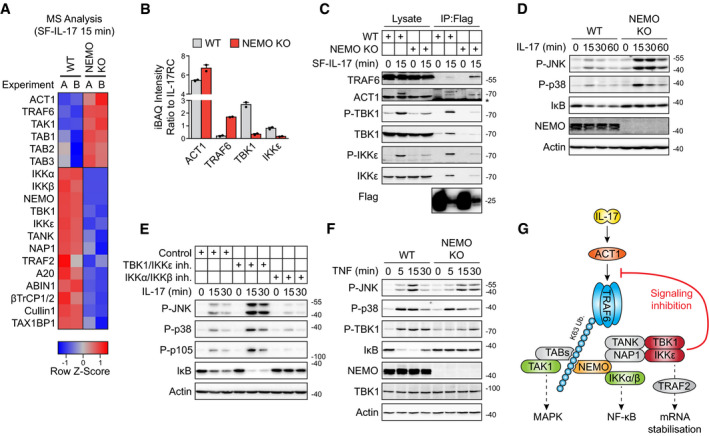
NEMO functions as an inhibitor of IL‐17RSC assembly A, BST2 wild‐type or NEMO KO cells were stimulated for 15 min with SF‐IL-17 (500 ng/ml), solubilized and IL‐17RSC was isolated via consecutive Flag and Strep immunoprecipitation and analyzed by MS. (A) The heat map shows the row‐normalized Z‐Score calculated from log_2_ transformed iBAQ values from two independent experiments. (B) The ratio between iBAQ intensities of selected IL‐17RSC components to iBAQ intensity of IL‐17RC. Mean + SEM from two independent experiments is shown.C, DST2 wild‐type or NEMO KO cells were stimulated with SF‐IL-17 (500 ng/ml) as indicated or were left unstimulated and IL‐17 was added post‐lysis. Lysates were subjected to anti‐Flag immunoprecipitation to isolate IL‐17RSC (C) or tested for activation of signaling pathways (D) and analyzed by immunoblotting.EST2 cells were pretreated with TBK1/IKKε inhibitor MRT67307 (2 μM) or IKKα/IKKβ inhibitor TPCA1 (10 μM), stimulated with IL‐17 (500 ng/ml) as indicated and analyzed by immunoblotting.FST2 wild‐type or NEMO KO cells were stimulated with TNF (50 ng/ml) for indicated time points and lysates were analyzed by immunoblotting.GSchematic model of the negative feedback loop mediated by NEMO‐recruited TBK1/IKKε.Immunoblot results are representative of four (C, D), three (E), or two (F) independent experiments.Source data are available online for this figure.

An interesting prediction of our model of proximal IL‐17 signaling is that NEMO has a dual role in the IL‐17 signaling as it acts both as a positive regulator by recruiting IKKα/β effector kinases and a negative regulator by recruiting TBK1/IKKε to the IL‐17RSC. To test this hypothesis, we compared the IL‐17RSC composition in WT and NEMO KO cells via MS (Table EV5). NEMO deficiency dramatically changed the composition of IL‐17RSC (Fig EV5A). In NEMO KO cells, the kinases IKKα and IKKβ were absent from IL‐17RSC, while the recruitment of TBK1 and IKKε was substantially decreased. Moreover, these cells exhibited strongly enhanced recruitment of TRAF6 (8.4‐fold) and TAB/TAK1 complex to the IL‐17RSC (Figs 5A–C and EV5B). In contrast, ACT1 recruitment was only very slightly increased (1.2‐fold), although its phosphorylation was not detectable (Fig 5B and C). Reconstitution of NEMO KO cells with NEMO(WT) enabled the recruitment of phosphorylated TBK1 and IKKε to the IL‐17RSC, leading to the phosphorylation of ACT1 and decreased recruitment of TRAF6, similarly to WT cells. In contrast, NEMO(Δ201–248) lacking the TANK/NAP1 interaction site was unable to rescue the phenotype (Fig EV5C).

Subsequently, we analyzed the IL‐17 signaling pathways in WT and NEMO KO cells. Whereas the NF‐κB pathway was abolished, the activation of MAPKs, JNK, and p38 was strongly enhanced in NEMO KO cells compared to WT cells (Fig 5D). In accord, the reconstitution of NEMO KO cells with NEMO (WT), but not with NEMO (Δ201–248) or an empty vector, inhibited the activation of the MAPK pathway by IL‐17 (Fig EV5D). Moreover, the inhibition of TBK1 and IKKε had no effect in NEMO‐deficient cells, demonstrating that NEMO is critical for the function of these kinases in IL‐17 signaling (Fig EV5E). We confirmed that NEMO negatively regulates the recruitment of TRAF6 into the IL‐17RSC and the IL‐17‐triggered MAPK activation also in human HeLa cells (Fig EV5F–H).

The negative role of NEMO in the IL‐17‐induced MAPK activation was independent of its well established role in the IKKα and IKKβ activation, because the chemical inhibition of these two kinases had no effect on the JNK and p38 activation (Fig 5E). Importantly, NEMO deficiency does not enhance the activation of JNK and p38 upon stimulation with TNF (Fig 5F). Altogether, our experiments identified a unique negative feedback loop in the proximal IL‐17 signaling pathway, represented by NEMO‐mediated recruitment of TBK1 and IKKε to the IL17‐RSC causing a release of the key ubiquitin ligase TRAF6 from the complex (Fig 5G).

### Phosphorylation of ACT1 by NEMO‐recruited TBK1 and IKKε on multiple residues inhibits TRAF6 recruitment

**Figure EV6:**
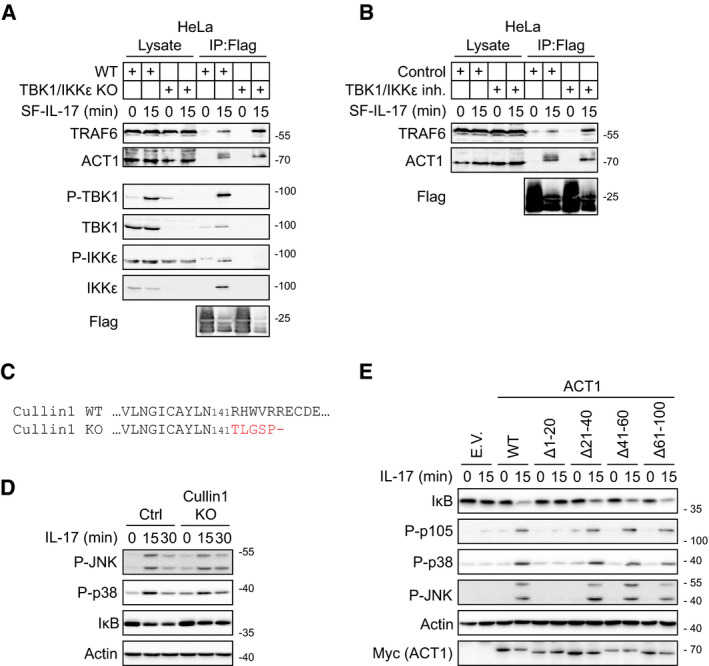
Activity of TBK1 and IKKε kinases promotes release of TRAF6 from the IL‐17RSC HeLa wild‐type or TBK1/IKKε DKO cells were stimulated with SF‐IL-17 as indicated, solubilized, and subjected to anti‐Flag immunoprecipitation to isolate IL‐17RSC. Samples were analyzed by immunoblotting.HeLa cells were treated or not with TBK1/IKKε inhibitor MRT67307 (2 μM) and stimulated with SF‐IL-17 (500 ng/ml) as indicated. IL‐17RSC was isolated by anti‐Flag immunoprecipitation and analyzed by immunoblotting.Cullin1 KO ST2 cells were prepared by CRISPR/Cas9 and verified by sequencing. Biallelic deletion of 2 base pairs leads to frameshift in Cullin1 coding sequence and premature STOP codon.Cullin1 KO ST2 cells were stimulated for 15 min with IL‐17 (500 ng/ml), and lysates were analyzed by immunoblotting.ACT1‐deficient ST2 cells were reconstituted with ACT1(WT) or ACT1 harboring indicated deletions or empty vector. Cells were stimulated for 15 min with IL‐17 (500 ng/ml), and lysates were analyzed by immunoblotting.Data information: Data are representative of two (A, B, E) or four (D) independent experiments.Source data are available online for this figure.

The absence of NEMO or both TBK1 and IKKε does not affect the recruitment of ACT1, but increases the amount of TRAF6 in the IL‐17RSC both in murine and human cells (Figs 3, 4, 5 and EV6A). Accordingly, the chemical inhibition of TBK1 and IKKε enhanced TRAF6 recruitment to the IL‐17RSC (Figs 3F and EV6B). TBK1/IKKε activity correlates with ACT1 phosphorylation upon IL‐17 stimulation (Figs 3C, 4B, 4D, and 5C and EV5C). ACT1 was shown to be degraded upon prolonged IL‐17 stimulation via Cullin1‐dependent mechanism (Shi *et al*, 2011). Although our data show that ablation of TBK1/IKKε leads to decreased recruitment of Cullin1 to IL‐17RSC (Figs 3A and EV3B), we did not observe increased phosphorylation of p38 and JNK in Cullin1 KO cells upon IL‐17 stimulation, even though Cullin1‐mediated degradation of IκB was inhibited (Fig EV6C and D). Therefore, a different mechanism inhibiting TRAF6 recruitment to IL‐17RSC must be responsible for the inhibitory effect of TBK1/IKKε‐mediated ACT1 phosphorylation.

**Figure 6:**
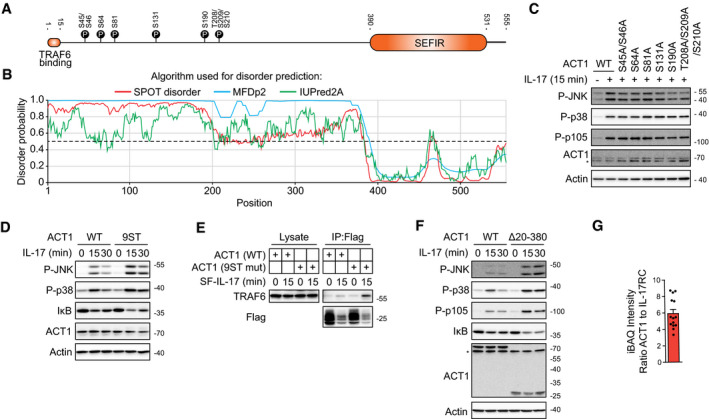
Phosphorylation of ACT1 in disordered region leads to the release of TRAF6 from IL‐17RSC ASchematic representation of ACT1 with indicated TRAF6‐binding domain, SEFIR domain and location of phosphorylation sites identified in our MS analysis (based on Table EV6).BAnalysis of ACT1 structure using three different algorithms predicting disordered regions. Detected phosphorylation sites are all in the disordered region of the protein.C, DACT1 KO cells were reconstituted with either wild‐type ACT1 or indicated mutants or ACT1 with all nine identified phospho‐sites mutated to alanines (9ST mut). Cells were stimulated with IL‐17 (500 ng/ml) as indicated and lysates were analyzed by immunoblotting. *indicates nonspecific band.EACT1 KO cells expressing either ACT1(WT) or ACT1(9ST mut) were stimulated with SF‐IL-17 (500 ng/ml) as indicated or were left unstimulated and IL‐17 was added post‐lysis. IL‐17RSC was isolated via anti‐Flag immunoprecipitation and analyzed by immunoblotting.FACT1 KO cells were reconstituted with either ACT1(WT) or ACT1 (Δ20–380). Cells were stimulated with IL‐17 (500 ng/ml) as indicated and lysates were analyzed by immunoblotting. *indicates nonspecific band.GAnalysis of iBAQ intensity of ACT1 normalized to iBAQ intensity of IL‐17RC. Data are composite of all 14 MS analyses of IL‐17RSC from wild‐type ST2 cells performed in this study. Mean + SEM is shown.Data information: Immunoblot results are representative of two (C, E) or four (D, F) independent experiments.Source data are available online for this figure.

ACT1 associates with IL‐17R via its C‐terminal SEFIR domain (Chang *et al*, 2006; Liu *et al*, 2011) and interacts with TRAF6 via its first 15 N‐terminal amino acids (Sonder *et al*, 2011) (Fig 6A). In accord, the reconstitution of ACT1‐deficient cells with a series of deletion mutants demonstrated that deletion of first 20 amino acids of ACT1 completely prevented IL‐17 signaling (Fig EV6E). Interestingly, the analysis of ACT1 structure *in silico* (Mizianty *et al*, 2013; Hanson *et al*, 2017; Meszaros *et al*, 2018) indicated that the mid‐part separating TRAF6‐binding site and the SEFIR domain is highly disordered (Fig 6B). Our MS data revealed that ACT1 in the IL‐17RSC was phosphorylated at multiple sites, all of them being within the unstructured mid‐part (Fig 6A and Table EV6). Furthermore, none of the phospho‐sites is located in the close proximity to the TRAF6‐binding domain, raising a question how these phosphorylation events affect TRAF6 recruitment. The reconstitution of ACT1‐deficient cells with ACT1 protein mutated in individual phospho‐sites to alanines did not affect the signaling outcome (Fig 6C), suggesting that there is not a single critical phospho‐site in ACT1. However, mutation of all these phospho‐sites substantially increased signaling responses to IL‐17 (Fig 6D) and strongly enhanced recruitment of TRAF6 to IL‐17RSC (Fig 6E). Altogether, these data showed that TBK1 and IKKε phosphorylate ACT1 at multiple sites in the disordered mid‐part of the protein to limit the amount of recruited TRAF6. In accord, reconstitution of ACT1 KO cells with ACT1 (Δ20–380) deletion mutant led to markedly enhanced proximal signaling as compared to ACT1(WT) (Fig 6F).

**Figure 7:**
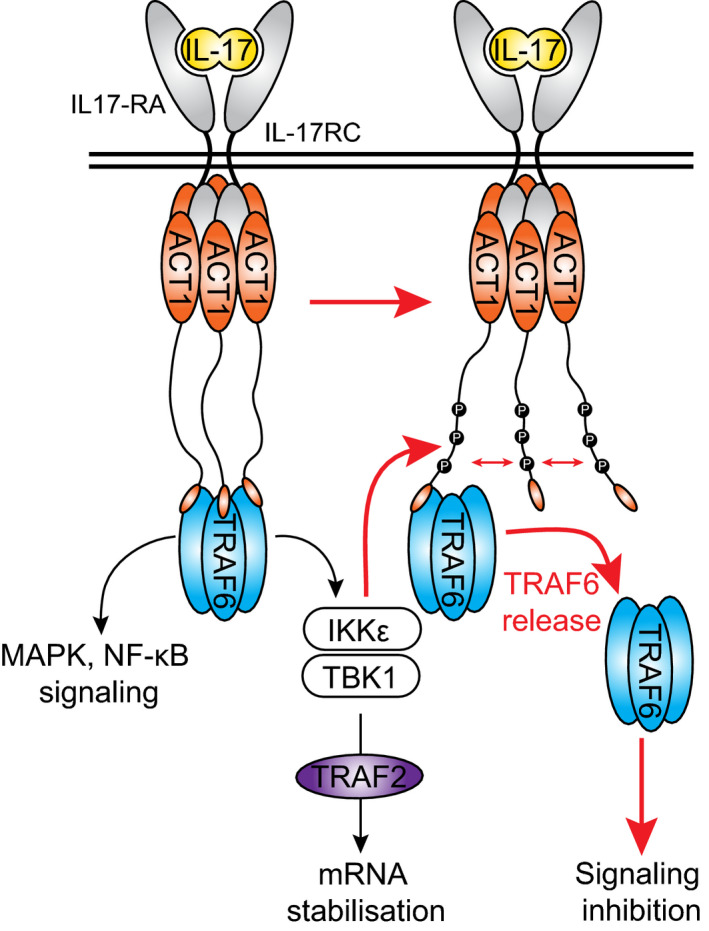
Schematic representation of the negative feedback loop modulating the assembly of IL‐17RSC Upon binding of IL‐17 to IL‐17RA and IL‐17RC, 6 molecules of ACT1 are recruited, creating docking site for trimeric TRAF6. TRAF6‐created K63‐polyubiquitin linkages promote activation of signaling pathways, but also trigger recruitment of TBK1 and IKKε kinases to IL‐17RSC. Both kinases then phosphorylate ACT1 in disordered region of the protein. This leads to a spatial separation of TRAF6‐binding sites present on ACT1, decreased avidity for TRAF6 and its release from IL‐17RSC, ultimately leading to inhibition of signaling. In addition, TBK1/IKKε enables recruitment of TRAF2 in kinase activity‐independent manner to promote stabilization of mRNA of target cytokines.

Based on these data, we propose a new model of IL‐17RSC formation and regulation. Triggering of the IL‐17RA/IL‐17RC by dimeric IL‐17 leads to the recruitment of approximately six ACT1 molecules as evident from our MS analysis (Fig 6G). These accumulated ACT1 molecules provide multiple TRAF6‐binding sites, generating a high‐avidity docking site for TRAF6 trimers (Ye *et al*, 2002). TRAF6‐mediated K63‐ubiquitin chains promote the recruitment and activation of effector molecules and kinases TBK1 and IKKε via NEMO. TBK1/IKKε have dual role: on one hand, they recruit TRAF2 in a kinase activity‐independent manner that is not required for activation of proximal signaling pathways, but promotes expression of target genes by stabilization of their transcripts. On the other hand, TBK1 and IKKε phosphorylate ACT1 at multiple sites within its unstructured mid‐part separating SEFIR‐ and TRAF6‐binding domains. This results in a strong negative charge‐mediated repulsion between individual ACT1 molecules. Subsequently, individual TRAF6‐binding domains are separated leading to the release of TRAF6 trimers from the complex (Fig 7). In this manner, NEMO‐mediated recruitment of TBK1 and IKKε kinases provide a potent negative feedback loop, which limits the IL‐17 signaling pathway.

## Discussion

Proper regulation of IL‐17RSC assembly and signaling is crucial for efficient immune responses while preventing autoimmunity. Our study provides a new methodological approach to analyze the assembly of the early signaling complex formed immediately upon binding of IL‐17 to its receptors. Based on this methodology, we identified two kinases, TBK1 and IKKε, as major components of the IL‐17RSC, whose recruitment is over 50‐fold increased when compared with related kinases IKKα and IKKβ. We demonstrated that these kinases are very strongly activated upon IL‐17 stimulation, to levels comparable with strongly proinflammatory cytokines IL‐1α or TNF.

The role of these kinases in IL‐17 signaling has been very controversial. Two highly influential studies showed that the ablation of either TBK1 or IKKε alone inhibited IL‐17 transcriptional responses (Bulek *et al*, 2011; Herjan *et al*, 2018). These articles promoted the view that these kinases have an activation role (Monin & Gaffen, 2018; Li *et al*, 2019; McGeachy *et al*, 2019; Swaidani *et al*, 2019). In accord, a recent publication analyzing the transcriptional response of cells upon IL‐17 stimulation in the presence of TNF demonstrated that deficiency of TBK1/IKKε led to markedly low activation of transcription, again supporting the view that these kinases propagate IL‐17 signaling (Tanaka *et al*, 2019). However, cells deficient in TBK1/IKKε are highly prone to TNF‐induced cell death (Bonnard *et al*, 2000; Matsui *et al*, 2006; Lafont *et al*, 2018; Xu *et al*, 2018) and it has been shown that mouse embryonic fibroblast deficient in TBK1/IKKε, used by Tanaka *et al*, rapidly succumb to TNF‐induced apoptosis and necroptosis (Lafont *et al*, 2018). Therefore, the irresponsiveness of TBK1/IKKε DKO cells to a combined IL‐17 and TNF treatment can be explained by their death.

On the other hand, another study described that overexpression of TBK1 or IKKε in TBK1/IKKε DKO cells inhibits the IL‐17 signaling via reducing the recruitment of TRAF6 to ACT1 (Qu *et al*, 2012). However, these experiments relied on overexpression systems and the molecular mechanism explaining the observed phenotype was only partially uncovered. Perhaps, this is why this study is either ignored or misinterpreted in a number of recent reviews on the topic of IL‐17 signaling (Gaffen *et al*, 2014; Monin & Gaffen, 2018; Li *et al*, 2019; McGeachy *et al*, 2019; Swaidani *et al*, 2019). In this study, we resolved the controversy and developed a model explaining the role of TBK1 and IKKε in regulation of IL‐17RSC assembly and signaling.

The core of IL‐17RSC contains dimeric IL‐17, one IL‐17RA and one IL‐17RC subunits (Ely *et al*, 2009; Liu *et al*, 2013; Goepfert *et al*, 2017). Our data demonstrated that this complex then recruits on average six ACT1 molecules. The molecular basis for ACT1 enrichment is presently unclear. ACT1 was described to form homo‐oligomers (Mauro *et al*, 2003; Liu *et al*, 2011; Boisson *et al*, 2013) and it is possible that once ACT1 is recruited, it can provide docking sites for additional ACT1 molecules. The enrichment of ACT1 proteins in the complex provides multiple binding sites for trimeric K63‐ubiquitin ligase TRAF6. Importantly, TRAF6 has relatively low affinity to monomeric scaffolds (Ye *et al*, 2002) and oligomerization of ACT1 likely enables high‐avidity interactions, which explains the molecular mechanism of TRAF6 enrichment at the IL‐17RSC. The recruitment of TRAF6 seems to be absolutely critical for IL‐17RSC assembly, since the ablation of TRAF6 or its enzymatic activity prevented recruitment of all other components of the complex. Analysis of various complexes dependent on formation of K63‐linkages for signaling showed a similar pattern of molecules that are recruited via binding to polyubiquitin linkages, including signaling complexes like TAB/TAK1 and NEMO/IKKα/IKKβ (Zinngrebe *et al*, 2014; Shimizu *et al*, 2015). However, these molecules are recruited very weakly to IL‐17, which correlates with poor signaling response of cells upon IL‐17 stimulation.

The canonical role of NEMO in IL‐17 and other signaling pathways is the recruitment and activation of IKKα/IKKβ kinases to trigger NF‐κB pathway (Hinz & Scheidereit, 2014). Here, we revealed an unexpected dual role of K63‐ubiquitin binding adaptor NEMO in IL‐17 signaling. NEMO recruits adaptors TANK and NAP1 which in turn bring two closely related kinases TBK1 and IKKε to IL‐17RSC, similarly to its role in TNF‐RSC (Clark *et al*, 2011b; Lafont *et al*, 2018). TBK1/IKKε are recruited to various signaling complexes, and depending on the particular context, they can modulate various cellular processes, ranging from the induction of mitophagy and autophagy (Wild *et al*, 2011; Heo *et al*, 2015; Thurston *et al*, 2016), interferon signaling (Fitzgerald *et al*, 2003; Sharma *et al*, 2003; Perry *et al*, 2004) to the protection from TNF‐induced cell death (Bonnard *et al*, 2000; Lafont *et al*, 2018; Xu *et al*, 2018).

Our data revealed that in the case of IL‐17RSC assembly, TBK1 and IKKε trigger a robust negative feedback loop by phosphorylating ACT1 on a number of residues in a long unstructured stretch of amino acids separating SEFIR domain and the TRAF6‐binding site. The sites identified in this study are partially different from previous reports (Bulek *et al*, 2011; Qu *et al*, 2012), which might reflect either differences in the experimental setup or in the cell lines used. However, our data indicate that phosphorylation of any of the phosphorylation sites within this part of ACT1 contributes to the spatial separation of TRAF6‐binding sites, leading to decreased avidity of TRAF6 for ACT1 oligomers and its release from the IL‐17RSC. This mechanism limits the ability of TRAF6 to create K63‐ubiquitin linkages and promote recruitment of additional effector molecules, but still ensures that some basal signaling is triggered. The inhibitory role of NEMO mediated by the recruitment of TBK1/IKKε is unprecedented as it has not been described previously and it currently seems to be unique for IL‐17. The ablation of the TBK1/IKKε‐mediated negative feedback loop completely changes the composition of the receptor by enhancing the recruitment of ubiquitin‐binding proteins, which allows the detection of signaling components such as LUBAC and TAB/TAK1. LUBAC amplifies signaling by recruiting additional NEMO/IKKα/IKKβ and enhancing NF‐κB activation (Kirisako *et al*, 2006; Haas *et al*, 2009; Tokunaga *et al*, 2009; Hrdinka & Gyrd‐Hansen, 2017), which is reflected in markedly enhanced transcription of IL‐17 target genes.

At the same time, TBK1/IKKε recruit TRAF2 in a kinase activity‐independent manner, which is required for the expression of IL‐17 responsive genes via stabilization of their transcripts (Sun *et al*, 2011; Herjan *et al*, 2013, 2018; Somma *et al*, 2015; Amatya *et al*, 2018). The exact molecular mechanism of how TRAF2 is recruited to the IL‐17RSC remains to be resolved, although both TBK1 and IKKε were shown previously to associate with TRAF2 (Pomerantz & Baltimore, 1999; Bonnard *et al*, 2000; Shen *et al*, 2012). Interestingly, we did not detect TRAF5, another reported regulator of mRNA stability (Sun *et al*, 2011), in the IL‐17RSC. One possibility is that the recruitment of TRAF5 to the complex might be weak or transient and below the detection limit of our method. Altogether, TBK1/IKKε kinases are major regulators of the complex and modulate its function. They inhibit recruitment of TRAF6 and therefore induction of signaling, while they promote recruitment of TRAF2 to promote expression of IL‐17 responsive genes. These results reconcile various seemingly contradictory findings concerning the role of TBK1/IKKε as both activators and inhibitors of signaling. The present biochemical data were not yet confirmed using *in vivo* animal models. However, the results presented in this work showing that inhibition of TBK1/IKKε leads to massively enhanced IL‐17‐induced signaling in both human and murine cell lines argue against potential therapeutic targeting of TBK1/IKKε kinase activity in treatment of IL‐17 mediated autoimmune diseases.

Our data identified a molecular mechanism for activation and regulation of IL‐17 signaling. The NEMO‐TBK1/IKKε‐mediated negative feedback loop allows only weak transcriptional response to IL‐17 stimulation. Given the major role of IL‐17 in orchestrating the function of the immune system, the tight control of IL‐17‐induced signaling via the NEMO‐TBK1/IKKε axis contributes to balancing the efficient immune response to pathogens and self‐tolerance.

## Materials and Methods

### Cell lines, reagents, and antibodies

ST2 cells were kindly provided by Jana Balounova (Institute of Molecular Genetics, Prague). HeLa, Hek293, and ØNX‐Eco, and ØNX‐Ampho cells were kindly provided by Tomas Brdicka (Institute of Molecular Genetics, Prague). Cells were cultivated in DMEM supplemented with 10% fetal calf serum (FCS; Gibco), 100 U/ml penicillin (BB Pharma), 100 μg/ml streptomycin (Sigma‐Aldrich), and 40 μg/ml gentamicin (Sandoz). Cells were regularly tested for mycoplasma contamination. Murine and human IL‐1α and murine TNF were purchased from Peprotech. Human TNF was produced as described previously (Draber *et al*, 2015). Inhibitors MRT67307, 7‐oxozeanol, and TPCA‐1 were from Tocris.

Primary antibodies used in this study were purchased from Cell Signaling: TBK1 (Cat#3013), P‐TBK1(S172) (D52C2, Cat#5483), murine IKKε (D61F9, Cat#3416), human IKKε (D20G4, Cat#2905), P‐IKKε (S172) (D1B7, Cat#8766), P‐IKKα/β (S176/180) (16A6, Cat#2697), IκB (Cat#9242), P‐IκB (Ser32/36) (5A5, Cat#9246), P‐p105 (Ser933) (18E6, Cat#4806), P‐p38 (Thr180/Tyr182) (D3F9, Cat#4511), P‐JNK (Thr183/Tyr185) (98F2, Cat#4671), P‐ATF2 (Thr71) (Cat#9221), P‐p65 (Ser536) (93H1, Cat#3033), TANK (Cat#2141), Myc (9B11, Cat#2276), TRAF2(C192, Cat#4724); from Abcam: NAP1/AZI2 (EPR14698, Cat#ab192253), TRAF6 (EP592Y, Cat#ab40675); from Santa Cruz: ACT1 (D‐11, Cat#sc‐398161), NEMO (FL‐419, Cat#sc‐8330); from Sigma: Flag (M2, Cat#F3165), Actin (AC‐15, Cat#A1978); from MRC PPU: HOIP (S174D); HOIL1 (S150D); from ProteinTech: Sharpin (Cat#14626‐I‐AP), and from BioLegend: Flag‐APC (L5, Cat#637307).

### Generation of knockout cell lines

In order to generate knockout cell lines via CRISPR/Cas9 approach, single‐guided RNA (sgRNA) targeting selected genes were designed using web tool CHOPCHOP (Labun *et al*, 2019) and inserted in pSpCas9(BB)‐2A‐GFP (PX458) vector kindly provided by Feng Zhang (Addgene plasmid #48138) (Ran *et al*, 2013). All constructs were sequenced. Below is the list of sgRNA target sequences with highlighted PAM motif (in bold and underlined) used to knockout different murine or human proteins:

Mouse TBK1 KO: 5′‐GGAAGTCCATACGCATTGGA**CGG**
Mouse IKKε KO: 5′‐GGCTGGCATGAACCACCTGC**GGG**
Mouse IKKε in TBK1/IKKε DKO: 5′‐GGGCCCACCGAAGGGGATGA**AGG**
Mouse HOIP KO: 5′‐GATGGATTGAGTTTCCCCGA**AGG**
Mouse ACT1 KO: 5′‐GTGGCCAAGAGATGATGCCC**CGG**
Mouse TRAF6 KO: 5′‐GCGTAAAGCCATCAAGCAGAT**GGG**
Mouse NEMO KO: 5′‐GTGCATTTCCAGGTCAGCCAG**CGG**
Mouse Cullin1 KO: 5′‐GTGCCTACCTCAATAGACAT**TGG**
Human TBK1 KO: 5′‐GGTAGTCCATAGGCATTAGA**AGG**
Human IKKε KO: 5′‐GAACATCATGCGCCTCGTAG**GGG**
Human NEMO KO: 5′‐GGCAGCAGATCAGGACGTAC**TGG**

Cells were transfected with PX458 vector containing particular sgRNA using Lipofectamine 2000 (Invitrogene) according to the manufacturer's instructions. Green fluorescent protein (GFP) expressing cells were sorted as single cell in 96‐well plates using FACSAria IIu (BD Biosciences). The resulting colonies were screened for the expression of target proteins by immunoblotting and sequencing of DNA surrounding the sgRNA target site.

### Gene expression using retroviral vectors

Coding sequences of different proteins were inserted in retroviral pBabe vector expressing GFP marker under SV40 promoter. Mutations in these sequences were performed by fusion PCR approach using Phusion polymerase (New England BioLabs). All constructs were sequenced.

For production of viruses, ØNX‐Eco cells (used for reconstitution of ST2 cells) or ØNX‐Ampho cells (used for reconstitution of HeLa cells) were transfected with pBabe‐GFP vector harboring coding sequences for indicated proteins or empty vector using Lipofectamine 2000. Virus containing supernatants were collected, passed through 0.2 μl filter and added to target cells in the presence of 6 μg/ml polybrene followed by spinning 1,200 *g* for 45 min. Infected cells were sorted as GFP positive using FACSAria IIu.

### Production and testing of recombinant SF‐IL‐17

DNA sequences coding the SF‐IL‐17 construct containing from N‐terminus: CD33 leader, 6xHis, 2xStrep tag, 1xFlag tag, and either murine IL‐17 (AA 26–158) or human IL‐17 (AA 24–155) were prepared using GeneArt Gene Synthesis service (Thermo Fisher Scientific) and inserted into pcDNA3.1 vector. These plasmids were transfected to adherent HEK293 cells using polyethylenimine (PEI) transfection; for transfection of the cells grown on one 15‐cm dish, we used 30 μg of the vector mixed with 75 μg PEI.

After 3 days, supernatants were collected and loaded on His GraviTrap TALON column (GE Healthcare) equilibrated in purification buffer (50 mM sodium phosphate pH 7.4, 300 mM NaCl). Columns were subsequently washed with 20 mM imidazole in purification buffer and eluted with 350 mM imidazole in purification buffer. In order to remove imidazol, samples were loaded on centrifugal filter (10 kDa molecular weight cutoff, Merck Millipore), washed several times with purification buffer and concentrated. The protein concentration was measured using NanoDrop (ND‐1000, Thermo Fisher Scientific). Recombinant proteins were mixed with equal volume of glycerol and kept at −80°C for long‐term storage.

In order to check the production of SF‐IL‐17, samples were mixed with SDS sample buffer, reduced with 50 mM dithiothreitol (DTT) or left untreated, separated using SDS‐PAGE and gels were stained with InstantBlue Coomassie protein stain (Expedeon).

In order to test functionality of produced SF‐IL‐17, cells were incubated on ice with SF‐IL‐17 (1 μg/ml) in FACS buffer (PBS/0.2%FCS/0.1%NaN_3_) and washed and binding of SF‐IL‐17 was detected using the APC‐conjugated anti‐Flag antibody on Accuri C6 cytometer (BD Biosciences) and analyzed by FlowJo (BD Biosciences).

### Cell stimulation

Prior to stimulation, ST2 or HeLa cells were washed and incubated in serum‐free DMEM medium for 30–60 min. In some experiments, cells were pretreated with indicated chemical inhibitor. Depending on whether murine or human cell line was used, cells were stimulated with murine or human SF‐IL‐17, IL‐1α, or TNF as indicated. Subsequently, cells were lysed in 1% *n*‐Dodecyl‐β‐d‐Maltoside (DDM) containing lysis buffer (30 mM Tris pH 7.4, 120 mM NaCl, 2 mM KCl, 2 mM EDTA, 10% glycerol, 10 mM chloroacetamide, 10 mM cOmplete protease inhibitor cocktail, and PhosSTOP tablets (Roche)). Samples were incubated at 4°C for 30 min, cleared by centrifugation (21,130 *g*, 30 min, 2°C), mixed with SDS sample buffer, reduced by 50 mM DTT, and analyzed by immunoblotting.

### Isolation of IL‐17RSC

For each experimental condition, ST2 or HeLa cells were grown on 15‐cm dish. Cells were washed and incubated 30–60 min in serum‐free DMEM. In some experiments, cells were pretreated with TBK1/IKKε inhibitor MRT67307 (2 μM). Cells were stimulated in 10 ml serum‐free DMEM with SF‐IL‐17 (500 ng/ml) for indicated time points, solubilized in 1.5 ml 1% DDM containing lysis buffer and cleared by centrifugation (21,130 *g*, 30 min, 2°C). In control samples, 0.5 μg of SF‐IL‐17 was added post‐lysis. Cleared lysates were incubated with 10 μl of anti‐FLAG M2 affinity agarose gel (Sigma) overnight, washed 3× with 0.1% DDM containing lysis buffer and eluted by boiling in SDS sample buffer with 50 mM DTT.

### Tandem affinity purification of IL‐17RSC

For each experimental condition, ST2 cells were grown on 6 × 15 cm dishes. Prior to stimulation, cells were washed and incubated in serum‐free DMEM for 30–60 min. Cells were activated with murine SF‐IL‐17 (500 ng/ml) for 15 min or left untreated. Subsequently, cells on each dish were solubilized in 1.5 ml of 1% DDM containing lysis buffer, collected, and incubated for 30 min on ice. In total, for each experimental condition 9 ml of lysates were obtained. Samples were cleared by centrifugation (21,130 *g*, 30 min, 2°C). In the control samples, 3 μg of SF‐IL‐17 was added post‐lysis.

The first immunoprecipitation step was carried out by overnight incubation of samples with 50 μl of anti‐FLAG M2 affinity agarose gel (Sigma). Subsequently, the beads were washed 3× with 0.1% DDM containing lysis buffer and isolated proteins were eluted by incubation of the beads in 250 μl of 1% DDM containing lysis buffer supplemented with 100 μg/ml of 3xFlag peptide (Sigma) overnight. The supernatant was collected and the elution step was repeated once again for 8 h.

The second purification step was carried upon incubation of the samples with 50 μl of Strep‐Tactin Sepharose beads (IBA Lifesciences) overnight. The samples were subsequently washed 3× with 0.1% DDM containing lysis buffer and 1× with lysis buffer alone. Bound proteins were eluted upon incubation of the beads with 220 μl of MS Elution buffer (2% sodium deoxycholate in 50 mM Tris pH 8.5).

### Protein digestion

The eluted protein samples (200 μl) were reduced with 5 mM tris(2‐carboxyethyl)phosphine at 60°C for 60 min and alkylated with 10 mM methyl methanethiosulfonate at room temperature for 10 min. Proteins were cleaved overnight with 1 μg of trypsin (Promega) at 37°C. In order to remove sodium deoxycholate, samples were acidified with 1% trifluoroacetic acid, mixed with equal volume of ethyl acetate, and centrifuged (15,700 *g*, 2 min) and aqueous phase containing peptides was collected (Masuda *et al*, 2008). This step was repeated for two more times. Peptides were desalted using in‐house made stage tips packed with C18 disks (Empore) (Rappsilber *et al*, 2007) and resuspended in 20 μl of 2% acetonitrile with 1% trifluoroacetic acid.

### nLC‐MS/MS analysis

The digested protein samples (12 μl) were loaded onto the trap column (Acclaim PepMap 300, C18, 5 μm, 300 Å Wide Pore, 300 μm × 5 mm) using 2% acetonitrile with 0.1% trifluoroacetic acid at a flow rate of 15 μl/min for 4 min. Subsequently, peptides were separated on Nano Reversed phase column (EASY‐Spray column, 50 cm × 75 μm internal diameter, packed with PepMap C18, 2 μm particles, 100 Å pore size) using linear gradient from 4 to 35% acetonitrile containing 0.1% formic acid at a flow rate of 300 nl/min for 60 min.

Ionized peptides were analyzed on a Thermo Orbitrap Fusion (Q‐OT‐qIT; Thermo Scientific). Survey scans of peptide precursors from 350 to 1,400 *m*/*z* were performed at 120K resolution settings with a 4 × 10^5^ ion count target. Four different types of tandem MS were performed according to precursor intensity. First three types were detected in Ion trap in rapid mode, and last one was detected in Orbitrap with 15,000 resolution settings: (i) For precursors with intensity between 1 × 10^3^ to 7 × 10^3^ with CID fragmentation (35% collision energy) and 250 ms of ion injection time. (ii) For ions with intensity in range from 7 × 10^3^ to 9 × 10^4^ with CID fragmentation (35% collision energy) and 100 ms of ion injection time. (iii) For ions with intensity in range from 9 × 10^4^ to 5 × 10^6^ with HCD fragmentation (30% collision energy) and 100 ms of ion injection time. (iv) For intensities 5 × 10^6^ and more with HCD fragmentation (30% collision energy) and 35 ms of ion injection time. The dynamic exclusion duration was set to 60 s with a 10 ppm tolerance around the selected precursor and its isotopes. Monoisotopic precursor selection was turned on. The instrument was run in top speed mode with 3 s cycles.

### Data analysis

All MS data were analyzed and quantified with the MaxQuant software (version 1.6.5.0) (Cox *et al*, 2014). The false discovery rate (FDR) was set to 1% for both proteins and peptides, and minimum length was specified as seven amino acids. The Andromeda search engine was used for the MS/MS spectra search against the murine Swiss‐Prot database (downloaded from Uniprot on June 2019). Trypsin specificity was set as C‐terminal to Arg and Lys, also allowing the cleavage at proline bonds and a maximum of two missed cleavages.

β‐methylthiolation, N‐terminal protein acetylation, carbamidomethylation, Met oxidation, and eventually Ser/Thr/Tyr phosphorylation, were included as variable modifications. Label‐free quantification was performed using intensity‐based absolute quantification (iBAQ) algorithm, which divides the sum of all precursor‐peptide intensities by the number of theoretically observable peptides (Schwanhausser *et al*, 2011). Data analysis was performed using Perseus 1.5.2.4 software (Tyanova *et al*, 2016).

Complex stoichiometry was estimated based on iBAQ intensity ratio between individual components of the complex and IL‐17RC. The PCAs and heat maps were constructed in programming language R. As source data, we used relative iBAQ values normalized to IL‐17RC with addition of pseudocount 0.001. These values were log2‐transformed prior to the analysis. The PCA was calculated using *prcomp* function. The heat map was constructed using *heatmap.2* function available in *gplots v3.0.1.1* package.

The analysis of ACT1 sequence to estimate the intrinsic disorder was performed using programs SPOT disorder (Hanson *et al*, 2017), MFDp2 (Mizianty *et al*, 2013), and IUPred2A (Meszaros *et al*, 2018).

### RNA sequencing experiments

For the RNA sequencing experiment, ST2 cells were grown in 6‐well plates, washed, and stimulated with IL‐17 (500 ng/ml) in the presence or absence of MRT67307 (2 μM) in DMEM supplemented with 0.5% FCS. Total RNA was extracted from the cells using RNeasy Mini Kit (Qiagen) with on column DNAse treatment exactly according the manufacturer′s protocol. The quality and quantity of isolated RNA were evaluated using Nanodrop and TapeStation 2200 (Agilent Technologies). The ensuing RNA sequencing analysis was based on three independent experiments.

For each sample, 1 μg of total RNA (RIN > 7.0, rRNA ratio [28S:18S rRNA] > 1.0) was sent to Macrogen Inc. for library preparation and sequencing. Briefly, first TruSeq RNA stranded library was generated and then sequencing was performed on the Illumina NovaSeq6000 with 100 bp paired‐ends configuration, with about 30M reads per sample. RNA was then quantified at the transcript‐level using Salmon (0.13.1) (Patro *et al*, 2017). First, a reference mouse transcriptome from Gencode M21(GRCm38.p6) was created by combining the sequence of protein‐coding genes and long non‐coding RNAs. Then, the transcriptome was indexed using Salmon index with parameter “–gencode”. Salmon quant was then run for each sample using parameters “–gcBias”, “–validateMappings”, and “–allowDovetail”.

Transcript‐level estimates from Salmon were summarized into gene level estimates using tximport (Soneson *et al*, 2015); all genes with at least 5 reads in at least 1 sample were retained for downstream analysis; with these parameters, 15,935 genes were obtained. Differential gene expression analysis was performed using DESeq2 (Love *et al*, 2014), using *independentFiltering* and using the Benjamini–Hochberg procedure to adjust *P*‐values. Genes with adjusted *P*‐value < 0.01 are reported as statistically significant.

### Quantitative real‐time PCR

Cells were washed and incubated 30–60 min in DMEM supplemented with 0.5% FCS. In some experiments, cells were pretreated with TBK1/IKKε inhibitor MRT67307 (2 μM). Cells were stimulated with SF‐IL‐17 (500 ng/ml) for 2 h and RNA was extracted with TRIzol (Thermo Fisher Scientific) and purified using RNA clean & concentrator kit (Zymo Research). Reverse transcription was performed with RevertAid Reverse Transcriptase (Thermo Fisher Scientific) using Oligo(dT)_18_ primers. Quantitative PCR was performed using SYBR Master Mix (Top‐Bio in Figs 2F and 4F or Roche in Figs EV2D and EV3F) or and analyzed on LightCycler 480 (Roche). Data were normalized to GAPDH. Following primer pairs for were used:

mGapdh: 5′‐TGCACCACCAACTGCTTAGC and 5′‐GGCATGGACTGTGGTCATGAG
mCxcl1: 5′‐CTTGAAGGTGTTGCCCTCAG and 5′‐TGGGGACACCTTTTAGCATC
mCxcl2: 5′‐CGGTCAAAAAGTTTGCCTTG and 5′‐TCCAGGTCAGTTAGCCTTGC
mTnf: 5′‐CCACCACGCTCTTCTGTCTAC and 5′‐AGGGTCTGGGCCATAGAACT
mIl6: 5′‐ATGGATGCTACCAAACTGGAT and 5′‐TGAAGGACTCTGGCTTTGTCT

### Statistics

The indicated statistical analyses were performed using Prism (GraphPad Software). The experimental data that were analyzed by unpaired two‐tailed Student's *t*‐test were tested for normality as follows: In Figs 1C, 2F, and EV3F data, normality was confirmed using Kolmogorov–Smirnov test. In Figs 4F and EV2D, normal distribution was assumed based on Fig 2F. In the case of Figs 3B and EV3B, in which certain proteins were not detected and thus did not follow normal distribution, two‐tailed nonparametric Mann–Whitney statistical tests were used.

## Author contributions

PD conceived the study. HD, SJ, DK, TS, MP, AU, AD, KR, OS and PD planned, performed, and analyzed experiments. SK, MH, VF, and GS performed RNA‐Seq experiment and analyzed data. KH contributed to design and analysis of MS measurements. PD and OS wrote the manuscript. All authors commented on the manuscript draft.

## Conflict of interest

The authors declare that they have no conflict of interest

## Supporting information



Expanded View Figures PDF

Table EV1

Table EV2

Table EV3

Table EV4

Table EV5

Table EV6

Source Data for Expanded View

Review Process File

Source Data for Figure 1

Source Data for Figure 2

Source Data for Figure 3

Source Data for Figure 4

Source Data for Figure 5

Source Data for Figure 6

## Data Availability

The datasets produced in this study are available in the following databases: The mass spectrometry data: PRIDE PXD019020 (http://www.ebi.ac.uk/pride/archive/projects/PXD019020). RNA‐Seq data: Gene Expression Omnibus GSE150410 (https://www.ncbi.nlm.nih.gov/geo/query/acc.cgi?acc=GSE150410).

## References

[embj2019104202-bib-0001] Amatya N , Garg AV , Gaffen SL (2017) IL‐17 signaling: the Yin and the Yang. Trends Immunol 38: 310–322 28254169 10.1016/j.it.2017.01.006 PMC5411326

[embj2019104202-bib-0002] Amatya N , Childs EE , Cruz JA , Aggor FEY , Garg AV , Berman AJ , Gudjonsson JE , Atasoy U , Gaffen SL (2018) IL‐17 integrates multiple self‐reinforcing, feed‐forward mechanisms through the RNA binding protein Arid5a. Sci Signal 11: eaat4617 30301788 10.1126/scisignal.aat4617 PMC6188668

[embj2019104202-bib-0003] Bilal J , Berlinberg A , Bhattacharjee S , Trost J , Riaz IB , Kurtzman DJB (2018) A systematic review and meta‐analysis of the efficacy and safety of the interleukin (IL)‐12/23 and IL‐17 inhibitors ustekinumab, secukinumab, ixekizumab, brodalumab, guselkumab and tildrakizumab for the treatment of moderate to severe plaque psoriasis. J Dermatolog Treat 29: 569–578 29532693 10.1080/09546634.2017.1422591

[embj2019104202-bib-0004] Boisson B , Wang C , Pedergnana V , Wu L , Cypowyj S , Rybojad M , Belkadi A , Picard C , Abel L , Fieschi C *et al* (2013) An ACT1 mutation selectively abolishes interleukin‐17 responses in humans with chronic mucocutaneous candidiasis. Immunity 39: 676–686 24120361 10.1016/j.immuni.2013.09.002 PMC3873857

[embj2019104202-bib-0005] Bonnard M , Mirtsos C , Suzuki S , Graham K , Huang J , Ng M , Itie A , Wakeham A , Shahinian A , Henzel WJ *et al* (2000) Deficiency of T2K leads to apoptotic liver degeneration and impaired NF‐kappaB‐dependent gene transcription. EMBO J 19: 4976–4985 10990461 10.1093/emboj/19.18.4976 PMC314216

[embj2019104202-bib-0006] Brembilla NC , Senra L , Boehncke WH (2018) The IL‐17 family of cytokines in psoriasis: IL‐17A and beyond. Front Immunol 9: 1682 30127781 10.3389/fimmu.2018.01682 PMC6088173

[embj2019104202-bib-0007] Bulek K , Liu C , Swaidani S , Wang L , Page RC , Gulen MF , Herjan T , Abbadi A , Qian W , Sun D *et al* (2011) The inducible kinase IKKi is required for IL‐17‐dependent signaling associated with neutrophilia and pulmonary inflammation. Nat Immunol 12: 844–852 21822257 10.1038/ni.2080 PMC3282992

[embj2019104202-bib-0008] Chang SH , Park H , Dong C (2006) Act1 adaptor protein is an immediate and essential signaling component of interleukin‐17 receptor. J Biol Chem 281: 35603–35607 17035243 10.1074/jbc.C600256200

[embj2019104202-bib-0009] Chariot A , Leonardi A , Muller J , Bonif M , Brown K , Siebenlist U (2002) Association of the adaptor TANK with the I kappa B kinase (IKK) regulator NEMO connects IKK complexes with IKK epsilon and TBK1 kinases. J Biol Chem 277: 37029–37036 12133833 10.1074/jbc.M205069200

[embj2019104202-bib-0010] Chau TL , Gioia R , Gatot JS , Patrascu F , Carpentier I , Chapelle JP , O'Neill L , Beyaert R , Piette J , Chariot A (2008) Are the IKKs and IKK‐related kinases TBK1 and IKK‐epsilon similarly activated? Trends Biochem Sci 33: 171–180 18353649 10.1016/j.tibs.2008.01.002

[embj2019104202-bib-0011] Cho JS , Pietras EM , Garcia NC , Ramos RI , Farzam DM , Monroe HR , Magorien JE , Blauvelt A , Kolls JK , Cheung AL *et al* (2010) IL‐17 is essential for host defense against cutaneous *Staphylococcus aureus* infection in mice. J Clin Invest 120: 1762–1773 20364087 10.1172/JCI40891 PMC2860944

[embj2019104202-bib-0012] Clark K , Peggie M , Plater L , Sorcek RJ , Young ER , Madwed JB , Hough J , McIver EG , Cohen P (2011a) Novel cross‐talk within the IKK family controls innate immunity. Biochem J 434: 93–104 21138416 10.1042/BJ20101701

[embj2019104202-bib-0013] Clark K , Takeuchi O , Akira S , Cohen P (2011b) The TRAF‐associated protein TANK facilitates cross‐talk within the IkappaB kinase family during Toll‐like receptor signaling. Proc Natl Acad Sci U S A 108: 17093–17098 21949249 10.1073/pnas.1114194108 PMC3193242

[embj2019104202-bib-0014] Conti HR , Shen F , Nayyar N , Stocum E , Sun JN , Lindemann MJ , Ho AW , Hai JH , Yu JJ , Jung JW *et al* (2009) Th17 cells and IL‐17 receptor signaling are essential for mucosal host defense against oral candidiasis. J Exp Med 206: 299–311 19204111 10.1084/jem.20081463 PMC2646568

[embj2019104202-bib-0015] Conti HR , Gaffen SL (2015) IL‐17‐mediated immunity to the opportunistic fungal pathogen *Candida albicans* . J Immunol 195: 780–788 26188072 10.4049/jimmunol.1500909 PMC4507294

[embj2019104202-bib-0016] Cox J , Hein MY , Luber CA , Paron I , Nagaraj N , Mann M (2014) Accurate proteome‐wide label‐free quantification by delayed normalization and maximal peptide ratio extraction, termed MaxLFQ. Mol Cell Proteomics 13: 2513–2526 24942700 10.1074/mcp.M113.031591 PMC4159666

[embj2019104202-bib-0017] Cua DJ , Tato CM (2010) Innate IL‐17‐producing cells: the sentinels of the immune system. Nat Rev Immunol 10: 479–489 20559326 10.1038/nri2800

[embj2019104202-bib-0018] Draber P , Kupka S , Reichert M , Draberova H , Lafont E , de Miguel D , Spilgies L , Surinova S , Taraborrelli L , Hartwig T *et al* (2015) LUBAC‐recruited CYLD and A20 regulate gene activation and cell death by exerting opposing effects on linear ubiquitin in signaling complexes. Cell Rep 13: 2258–2272 26670046 10.1016/j.celrep.2015.11.009 PMC4688036

[embj2019104202-bib-0019] Ea CK , Deng L , Xia ZP , Pineda G , Chen ZJ (2006) Activation of IKK by TNFalpha requires site‐specific ubiquitination of RIP1 and polyubiquitin binding by NEMO. Mol Cell 22: 245–257 16603398 10.1016/j.molcel.2006.03.026

[embj2019104202-bib-0020] Ely LK , Fischer S , Garcia KC (2009) Structural basis of receptor sharing by interleukin 17 cytokines. Nat Immunol 10: 1245–1251 19838198 10.1038/ni.1813 PMC2783927

[embj2019104202-bib-0021] Fitzgerald KA , McWhirter SM , Faia KL , Rowe DC , Latz E , Golenbock DT , Coyle AJ , Liao SM , Maniatis T (2003) IKKepsilon and TBK1 are essential components of the IRF3 signaling pathway. Nat Immunol 4: 491–496 12692549 10.1038/ni921

[embj2019104202-bib-0022] Gaffen SL , Jain R , Garg AV , Cua DJ (2014) The IL‐23‐IL‐17 immune axis: from mechanisms to therapeutic testing. Nat Rev Immunol 14: 585–600 25145755 10.1038/nri3707 PMC4281037

[embj2019104202-bib-0023] Garg AV , Ahmed M , Vallejo AN , Ma A , Gaffen SL (2013) The deubiquitinase A20 mediates feedback inhibition of interleukin‐17 receptor signaling. Sci Signal 6: ra44 23737552 10.1126/scisignal.2003699 PMC4028484

[embj2019104202-bib-0024] Gerlach B , Cordier SM , Schmukle AC , Emmerich CH , Rieser E , Haas TL , Webb AI , Rickard JA , Anderton H , Wong WW *et al* (2011) Linear ubiquitination prevents inflammation and regulates immune signalling. Nature 471: 591–596 21455173 10.1038/nature09816

[embj2019104202-bib-0025] Goepfert A , Lehmann S , Wirth E , Rondeau JM (2017) The human IL‐17A/F heterodimer: a two‐faced cytokine with unique receptor recognition properties. Sci Rep 7: 8906 28827714 10.1038/s41598-017-08360-9 PMC5566378

[embj2019104202-bib-0026] Haas TL , Emmerich CH , Gerlach B , Schmukle AC , Cordier SM , Rieser E , Feltham R , Vince J , Warnken U , Wenger T *et al* (2009) Recruitment of the linear ubiquitin chain assembly complex stabilizes the TNF‐R1 signaling complex and is required for TNF‐mediated gene induction. Mol Cell 36: 831–844 20005846 10.1016/j.molcel.2009.10.013

[embj2019104202-bib-0027] Hanson J , Yang Y , Paliwal K , Zhou Y (2017) Improving protein disorder prediction by deep bidirectional long short‐term memory recurrent neural networks. Bioinformatics 33: 685–692 28011771 10.1093/bioinformatics/btw678

[embj2019104202-bib-0028] Harrington LE , Hatton RD , Mangan PR , Turner H , Murphy TL , Murphy KM , Weaver CT (2005) Interleukin 17‐producing CD4^+^ effector T cells develop via a lineage distinct from the T helper type 1 and 2 lineages. Nat Immunol 6: 1123–1132 16200070 10.1038/ni1254

[embj2019104202-bib-0029] Hawkes JE , Yan BY , Chan TC , Krueger JG (2018) Discovery of the IL‐23/IL‐17 signaling pathway and the treatment of Psoriasis. J Immunol 201: 1605–1613 30181299 10.4049/jimmunol.1800013 PMC6129988

[embj2019104202-bib-0030] Helgason E , Phung QT , Dueber EC (2013) Recent insights into the complexity of Tank‐binding kinase 1 signaling networks: the emerging role of cellular localization in the activation and substrate specificity of TBK1. FEBS Lett 587: 1230–1237 23395801 10.1016/j.febslet.2013.01.059

[embj2019104202-bib-0031] Heo JM , Ordureau A , Paulo JA , Rinehart J , Harper JW (2015) The PINK1‐PARKIN mitochondrial ubiquitylation pathway drives a program of OPTN/NDP52 recruitment and TBK1 activation to promote mitophagy. Mol Cell 60: 7–20 26365381 10.1016/j.molcel.2015.08.016 PMC4592482

[embj2019104202-bib-0032] Herjan T , Yao P , Qian W , Li X , Liu C , Bulek K , Sun D , Yang WP , Zhu J , He A *et al* (2013) HuR is required for IL‐17‐induced Act1‐mediated CXCL1 and CXCL5 mRNA stabilization. J Immunol 191: 640–649 23772036 10.4049/jimmunol.1203315 PMC3722902

[embj2019104202-bib-0033] Herjan T , Hong L , Bubenik J , Bulek K , Qian W , Liu C , Li X , Chen X , Yang H , Ouyang S *et al* (2018) IL‐17‐receptor‐associated adaptor Act1 directly stabilizes mRNAs to mediate IL‐17 inflammatory signaling. Nat Immunol 19: 354–365 29563620 10.1038/s41590-018-0071-9 PMC6082628

[embj2019104202-bib-0034] Hinz M , Scheidereit C (2014) The IkappaB kinase complex in NF‐kappaB regulation and beyond. EMBO Rep 15: 46–61 24375677 10.1002/embr.201337983 PMC4303448

[embj2019104202-bib-0035] Hrdinka M , Gyrd‐Hansen M (2017) The Met1‐linked ubiquitin machinery: emerging themes of (De)regulation. Mol Cell 68: 265–280 29053955 10.1016/j.molcel.2017.09.001

[embj2019104202-bib-0036] Hu Y , Ota N , Peng I , Refino CJ , Danilenko DM , Caplazi P , Ouyang W (2010) IL‐17RC is required for IL‐17A‐ and IL‐17F‐dependent signaling and the pathogenesis of experimental autoimmune encephalomyelitis. J Immunol 184: 4307–4316 20231694 10.4049/jimmunol.0903614

[embj2019104202-bib-0037] Ikeda F , Deribe YL , Skanland SS , Stieglitz B , Grabbe C , Franz‐Wachtel M , van Wijk SJ , Goswami P , Nagy V , Terzic J *et al* (2011) SHARPIN forms a linear ubiquitin ligase complex regulating NF‐kappaB activity and apoptosis. Nature 471: 637–641 21455181 10.1038/nature09814 PMC3085511

[embj2019104202-bib-0038] Kirisako T , Kamei K , Murata S , Kato M , Fukumoto H , Kanie M , Sano S , Tokunaga F , Tanaka K , Iwai K (2006) A ubiquitin ligase complex assembles linear polyubiquitin chains. EMBO J 25: 4877–4887 17006537 10.1038/sj.emboj.7601360 PMC1618115

[embj2019104202-bib-0039] Kishore N , Huynh QK , Mathialagan S , Hall T , Rouw S , Creely D , Lange G , Caroll J , Reitz B , Donnelly A *et al* (2002) IKK‐i and TBK‐1 are enzymatically distinct from the homologous enzyme IKK‐2: comparative analysis of recombinant human IKK‐i, TBK‐1, and IKK‐2. J Biol Chem 277: 13840–13847 11839743 10.1074/jbc.M110474200

[embj2019104202-bib-0040] Kuestner RE , Taft DW , Haran A , Brandt CS , Brender T , Lum K , Harder B , Okada S , Ostrander CD , Kreindler JL *et al* (2007) Identification of the IL‐17 receptor related molecule IL‐17RC as the receptor for IL‐17F. J Immunol 179: 5462–5473 17911633 10.4049/jimmunol.179.8.5462 PMC2849293

[embj2019104202-bib-0041] Kupka S , Reichert M , Draber P , Walczak H (2016) Formation and removal of poly‐ubiquitin chains in the regulation of tumor necrosis factor‐induced gene activation and cell death. FEBS J 283: 2626–2639 26749412 10.1111/febs.13644

[embj2019104202-bib-0042] Labun K , Montague TG , Krause M , Torres Cleuren YN , Tjeldnes H , Valen E (2019) CHOPCHOP v3: expanding the CRISPR web toolbox beyond genome editing. Nucleic Acids Res 47: W171–W174 31106371 10.1093/nar/gkz365 PMC6602426

[embj2019104202-bib-0043] Lafont E , Draber P , Rieser E , Reichert M , Kupka S , de Miguel D , Draberova H , von Massenhausen A , Bhamra A , Henderson S *et al* (2018) TBK1 and IKKepsilon prevent TNF‐induced cell death by RIPK1 phosphorylation. Nat Cell Biol 20: 1389–1399 30420664 10.1038/s41556-018-0229-6 PMC6268100

[embj2019104202-bib-0044] Laplantine E , Fontan E , Chiaravalli J , Lopez T , Lakisic G , Veron M , Agou F , Israel A (2009) NEMO specifically recognizes K63‐linked poly‐ubiquitin chains through a new bipartite ubiquitin‐binding domain. EMBO J 28: 2885–2895 19763089 10.1038/emboj.2009.241 PMC2760117

[embj2019104202-bib-0045] Larabi A , Devos JM , Ng SL , Nanao MH , Round A , Maniatis T , Panne D (2013) Crystal structure and mechanism of activation of TANK‐binding kinase 1. Cell Rep 3: 734–746 23453971 10.1016/j.celrep.2013.01.034

[embj2019104202-bib-0046] Li X , Bechara R , Zhao J , McGeachy MJ , Gaffen SL (2019) IL‐17 receptor‐based signaling and implications for disease. Nat Immunol 20: 1594–1602 31745337 10.1038/s41590-019-0514-y PMC6943935

[embj2019104202-bib-0047] Liu C , Swaidani S , Qian W , Kang Z , Sun P , Han Y , Wang C , Gulen MF , Yin W , Zhang C *et al* (2011) A CC’ loop decoy peptide blocks the interaction between Act1 and IL‐17RA to attenuate IL‐17‐ and IL‐25‐induced inflammation. Sci Signal 4: ra72 22045852 10.1126/scisignal.2001843 PMC3282585

[embj2019104202-bib-0048] Liu S , Song X , Chrunyk BA , Shanker S , Hoth LR , Marr ES , Griffor MC (2013) Crystal structures of interleukin 17A and its complex with IL‐17 receptor A. Nat Commun 4: 1888 23695682 10.1038/ncomms2880

[embj2019104202-bib-0049] Love MI , Huber W , Anders S (2014) Moderated estimation of fold change and dispersion for RNA‐seq data with DESeq2. Genome Biol 15: 550 25516281 10.1186/s13059-014-0550-8 PMC4302049

[embj2019104202-bib-0050] Ma X , Helgason E , Phung QT , Quan CL , Iyer RS , Lee MW , Bowman KK , Starovasnik MA , Dueber EC (2012) Molecular basis of Tank‐binding kinase 1 activation by transautophosphorylation. Proc Natl Acad Sci U S A 109: 9378–9383 22619329 10.1073/pnas.1121552109 PMC3386122

[embj2019104202-bib-0051] Masuda T , Tomita M , Ishihama Y (2008) Phase transfer surfactant‐aided trypsin digestion for membrane proteome analysis. J Proteome Res 7: 731–740 18183947 10.1021/pr700658q

[embj2019104202-bib-0052] Matsui K , Kumagai Y , Kato H , Sato S , Kawagoe T , Uematsu S , Takeuchi O , Akira S (2006) Cutting edge: role of TANK‐binding kinase 1 and inducible IkappaB kinase in IFN responses against viruses in innate immune cells. J Immunol 177: 5785–5789 17056502 10.4049/jimmunol.177.9.5785

[embj2019104202-bib-0053] Mauro C , Vito P , Mellone S , Pacifico F , Chariot A , Formisano S , Leonardi A (2003) Role of the adaptor protein CIKS in the activation of the IKK complex. Biochem Biophys Res Commun 309: 84–90 12943667 10.1016/s0006-291x(03)01532-8

[embj2019104202-bib-0054] McGeachy MJ , Cua DJ , Gaffen SL (2019) The IL‐17 family of cytokines in health and disease. Immunity 50: 892–906 30995505 10.1016/j.immuni.2019.03.021 PMC6474359

[embj2019104202-bib-0055] Meszaros B , Erdos G , Dosztanyi Z (2018) IUPred2A: context‐dependent prediction of protein disorder as a function of redox state and protein binding. Nucleic Acids Res 46: W329–W337 29860432 10.1093/nar/gky384 PMC6030935

[embj2019104202-bib-0056] Mizianty MJ , Peng Z , Kurgan L (2013) MFDp2: accurate predictor of disorder in proteins by fusion of disorder probabilities, content and profiles. Intrinsically Disord Proteins 1: e24428 28516009 10.4161/idp.24428 PMC5424793

[embj2019104202-bib-0057] Monin L , Gaffen SL (2018) Interleukin 17 family cytokines: signaling mechanisms, biological activities, and therapeutic implications. Cold Spring Harb Perspect Biol 10: a028522 28620097 10.1101/cshperspect.a028522 PMC5732092

[embj2019104202-bib-0058] Park H , Li Z , Yang XO , Chang SH , Nurieva R , Wang YH , Wang Y , Hood L , Zhu Z , Tian Q *et al* (2005) A distinct lineage of CD4 T cells regulates tissue inflammation by producing interleukin 17. Nat Immunol 6: 1133–1141 16200068 10.1038/ni1261 PMC1618871

[embj2019104202-bib-0059] Patro R , Duggal G , Love MI , Irizarry RA , Kingsford C (2017) Salmon provides fast and bias‐aware quantification of transcript expression. Nat Methods 14: 417–419 28263959 10.1038/nmeth.4197 PMC5600148

[embj2019104202-bib-0060] Perry AK , Chow EK , Goodnough JB , Yeh WC , Cheng G (2004) Differential requirement for TANK‐binding kinase‐1 in type I interferon responses to toll‐like receptor activation and viral infection. J Exp Med 199: 1651–1658 15210743 10.1084/jem.20040528 PMC2212814

[embj2019104202-bib-0061] Pomerantz JL , Baltimore D (1999) NF‐kappaB activation by a signaling complex containing TRAF2, TANK and TBK1, a novel IKK‐related kinase. EMBO J 18: 6694–6704 10581243 10.1093/emboj/18.23.6694 PMC1171732

[embj2019104202-bib-0062] Puel A , Cypowyj S , Bustamante J , Wright JF , Liu L , Lim HK , Migaud M , Israel L , Chrabieh M , Audry M *et al* (2011) Chronic mucocutaneous candidiasis in humans with inborn errors of interleukin‐17 immunity. Science 332: 65–68 21350122 10.1126/science.1200439 PMC3070042

[embj2019104202-bib-0063] Qian Y , Liu C , Hartupee J , Altuntas CZ , Gulen MF , Jane‐Wit D , Xiao J , Lu Y , Giltiay N , Liu J *et al* (2007) The adaptor Act1 is required for interleukin 17‐dependent signaling associated with autoimmune and inflammatory disease. Nat Immunol 8: 247–256 17277779 10.1038/ni1439

[embj2019104202-bib-0064] Qu F , Gao H , Zhu S , Shi P , Zhang Y , Liu Y , Jallal B , Yao Y , Shi Y , Qian Y (2012) TRAF6‐dependent Act1 phosphorylation by the IkappaB kinase‐related kinases suppresses interleukin‐17‐induced NF‐kappaB activation. Mol Cell Biol 32: 3925–3937 22851696 10.1128/MCB.00268-12 PMC3457531

[embj2019104202-bib-0065] Ran FA , Hsu PD , Wright J , Agarwala V , Scott DA , Zhang F (2013) Genome engineering using the CRISPR‐Cas9 system. Nat Protoc 8: 2281–2308 24157548 10.1038/nprot.2013.143 PMC3969860

[embj2019104202-bib-0066] Rappsilber J , Mann M , Ishihama Y (2007) Protocol for micro‐purification, enrichment, pre‐fractionation and storage of peptides for proteomics using StageTips. Nat Protoc 2: 1896–1906 17703201 10.1038/nprot.2007.261

[embj2019104202-bib-0067] Schwandner R , Yamaguchi K , Cao Z (2000) Requirement of tumor necrosis factor receptor‐associated factor (TRAF)6 in interleukin 17 signal transduction. J Exp Med 191: 1233–1240 10748240 10.1084/jem.191.7.1233 PMC2193168

[embj2019104202-bib-0068] Schwanhausser B , Busse D , Li N , Dittmar G , Schuchhardt J , Wolf J , Chen W , Selbach M (2011) Global quantification of mammalian gene expression control. Nature 473: 337–342 21593866 10.1038/nature10098

[embj2019104202-bib-0069] Sharma S , tenOever BR , Grandvaux N , Zhou GP , Lin R , Hiscott J (2003) Triggering the interferon antiviral response through an IKK‐related pathway. Science 300: 1148–1151 12702806 10.1126/science.1081315

[embj2019104202-bib-0070] Shen RR , Zhou AY , Kim E , Lim E , Habelhah H , Hahn WC (2012) IkappaB kinase epsilon phosphorylates TRAF2 to promote mammary epithelial cell transformation. Mol Cell Biol 32: 4756–4768 23007157 10.1128/MCB.00468-12 PMC3497603

[embj2019104202-bib-0071] Shi P , Zhu S , Lin Y , Liu Y , Liu Y , Chen Z , Shi Y , Qian Y (2011) Persistent stimulation with interleukin‐17 desensitizes cells through SCFbeta‐TrCP‐mediated degradation of Act1. Sci Signal 4: ra73 22045853 10.1126/scisignal.2001653

[embj2019104202-bib-0072] Shimizu Y , Taraborrelli L , Walczak H (2015) Linear ubiquitination in immunity. Immunol Rev 266: 190–207 26085216 10.1111/imr.12309 PMC4737190

[embj2019104202-bib-0073] Somma D , Mastrovito P , Grieco M , Lavorgna A , Pignalosa A , Formisano L , Salzano AM , Scaloni A , Pacifico F , Siebenlist U *et al* (2015) CIKS/DDX3X interaction controls the stability of the Zc3h12a mRNA induced by IL‐17. J Immunol 194: 3286–3294 25710910 10.4049/jimmunol.1401589 PMC4369453

[embj2019104202-bib-0074] Sonder SU , Saret S , Tang W , Sturdevant DE , Porcella SF , Siebenlist U (2011) IL‐17‐induced NF‐kappaB activation via CIKS/Act1: physiologic significance and signaling mechanisms. J Biol Chem 286: 12881–12890 21335551 10.1074/jbc.M110.199547 PMC3075635

[embj2019104202-bib-0075] Soneson C , Love MI , Robinson MD (2015) Differential analyses for RNA‐seq: transcript‐level estimates improve gene‐level inferences. F1000Res 4: 1521. 26925227 10.12688/f1000research.7563.1 PMC4712774

[embj2019104202-bib-0076] Strickson S , Emmerich CH , Goh ET , Zhang J , Kelsall IR , Macartney T , Hastie CJ , Knebel A , Peggie M , Marchesi F *et al* (2017) Roles of the TRAF6 and Pellino E3 ligases in MyD88 and RANKL signaling. Proc Natl Acad Sci U S A 114: E3481–E3489 28404732 10.1073/pnas.1702367114 PMC5410814

[embj2019104202-bib-0077] Sun D , Novotny M , Bulek K , Liu C , Li X , Hamilton T (2011) Treatment with IL‐17 prolongs the half‐life of chemokine CXCL1 mRNA via the adaptor TRAF5 and the splicing‐regulatory factor SF2 (ASF). Nat Immunol 12: 853–860 21822258 10.1038/ni.2081 PMC3597344

[embj2019104202-bib-0078] Swaidani S , Liu C , Zhao J , Bulek K , Li X (2019) TRAF regulation of IL‐17 cytokine signaling. Front Immunol 10: 1293 31316496 10.3389/fimmu.2019.01293 PMC6610456

[embj2019104202-bib-0079] Tanaka H , Arima Y , Kamimura D , Tanaka Y , Takahashi N , Uehata T , Maeda K , Satoh T , Murakami M , Akira S (2019) Phosphorylation‐dependent Regnase‐1 release from endoplasmic reticulum is critical in IL‐17 response. J Exp Med 216: 1431–1449 31072819 10.1084/jem.20181078 PMC6547859

[embj2019104202-bib-0080] Thurston TL , Boyle KB , Allen M , Ravenhill BJ , Karpiyevich M , Bloor S , Kaul A , Noad J , Foeglein A , Matthews SA *et al* (2016) Recruitment of TBK1 to cytosol‐invading Salmonella induces WIPI2‐dependent antibacterial autophagy. EMBO J 35: 1779–1792 27370208 10.15252/embj.201694491 PMC5010046

[embj2019104202-bib-0081] Tokunaga F , Sakata S , Saeki Y , Satomi Y , Kirisako T , Kamei K , Nakagawa T , Kato M , Murata S , Yamaoka S *et al* (2009) Involvement of linear polyubiquitylation of NEMO in NF‐kappaB activation. Nat Cell Biol 11: 123–132 19136968 10.1038/ncb1821

[embj2019104202-bib-0082] Tokunaga F , Nakagawa T , Nakahara M , Saeki Y , Taniguchi M , Sakata S , Tanaka K , Nakano H , Iwai K (2011) SHARPIN is a component of the NF‐kappaB‐activating linear ubiquitin chain assembly complex. Nature 471: 633–636 21455180 10.1038/nature09815

[embj2019104202-bib-0083] Toy D , Kugler D , Wolfson M , Vanden Bos T , Gurgel J , Derry J , Tocker J , Peschon J (2006) Cutting edge: interleukin 17 signals through a heteromeric receptor complex. J Immunol 177: 36–39 16785495 10.4049/jimmunol.177.1.36

[embj2019104202-bib-0084] Tyanova S , Temu T , Sinitcyn P , Carlson A , Hein MY , Geiger T , Mann M , Cox J (2016) The Perseus computational platform for comprehensive analysis of (prote)omics data. Nat Methods 13: 731–740 27348712 10.1038/nmeth.3901

[embj2019104202-bib-0085] Wild P , Farhan H , McEwan DG , Wagner S , Rogov VV , Brady NR , Richter B , Korac J , Waidmann O , Choudhary C *et al* (2011) Phosphorylation of the autophagy receptor optineurin restricts Salmonella growth. Science 333: 228–233 21617041 10.1126/science.1205405 PMC3714538

[embj2019104202-bib-0086] Wu CJ , Conze DB , Li T , Srinivasula SM , Ashwell JD (2006) Sensing of Lys 63‐linked polyubiquitination by NEMO is a key event in NF‐kappaB activation [corrected]. Nat Cell Biol 8: 398–406 16547522 10.1038/ncb1384

[embj2019104202-bib-0087] Xu D , Jin T , Zhu H , Chen H , Ofengeim D , Zou C , Mifflin L , Pan L , Amin P , Li W *et al* (2018) TBK1 suppresses RIPK1‐driven apoptosis and inflammation during development and in aging. Cell 174: 1477–1491.e1419 30146158 10.1016/j.cell.2018.07.041 PMC6128749

[embj2019104202-bib-0088] Ye H , Arron JR , Lamothe B , Cirilli M , Kobayashi T , Shevde NK , Segal D , Dzivenu OK , Vologodskaia M , Yim M *et al* (2002) Distinct molecular mechanism for initiating TRAF6 signalling. Nature 418: 443–447 12140561 10.1038/nature00888

[embj2019104202-bib-0089] Zinngrebe J , Montinaro A , Peltzer N , Walczak H (2014) Ubiquitin in the immune system. EMBO Rep 15: 28–45 24375678 10.1002/embr.201338025 PMC4303447

