# Supplementary material for: Systematic analysis of the IL‐17 receptor signalosome reveals a robust regulatory feedback loop
Source: EMBO J. 2020 Jul 21;39(17):e104202. doi: 10.15252/embj.2019104202 (PMC7459424; doi:10.15252/embj.2019104202)
Supplement: Supplementary file 1 — Expanded View Figures PDF [file EMBJ-39-e104202-s001.pdf]

Expanded View Figures

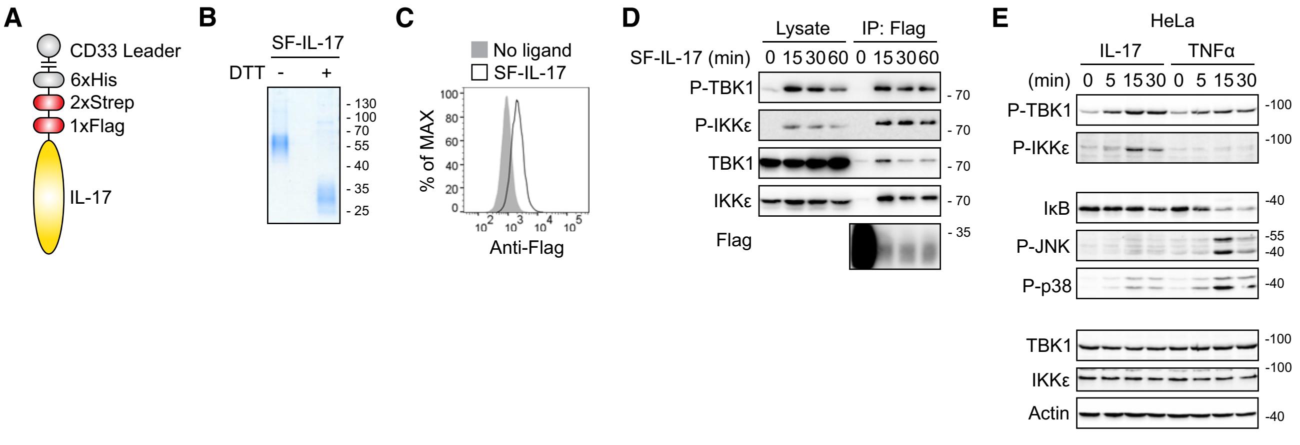

Figure EV1. Isolation of IL-17RSC reveals that TBK1 and IKKε kinases are strongly activated upon IL-17 stimulation.

A The schematic representation of recombinant Strep-Flag-IL-17 (SF-IL-17) construct used in this study. Murine or human IL-17 coding sequence lacking leader peptide was used.

B The purity and assembly of SF-IL-17 was analyzed by SDS-PAGE followed by Coomassie staining. Samples were either left untreated or reduced with dithiothreitol (DTT) to disrupt the covalent IL-17 dimers.

C ST2 cells were incubated on ice in the presence or absence of SF-IL-17. Subsequently, the cells were stained with fluorescently labeled anti-Flag antibody and analyzed by FACS.

D ST2 cells were stimulated with SF-IL-17 (500 ng/ml) for indicated time points or were left unstimulated and IL-17 was added post-lysis. Lysates were subjected to anti-Flag immunoprecitation to isolate IL-17RSC. The samples were analyzed by immunoblotting.

E HeLa cells were stimulated with IL-17 (500 ng/ml) or TNF (500 ng/ml) for indicated time points and analyzed via immunoblotting.

Source data are available online for this figure.

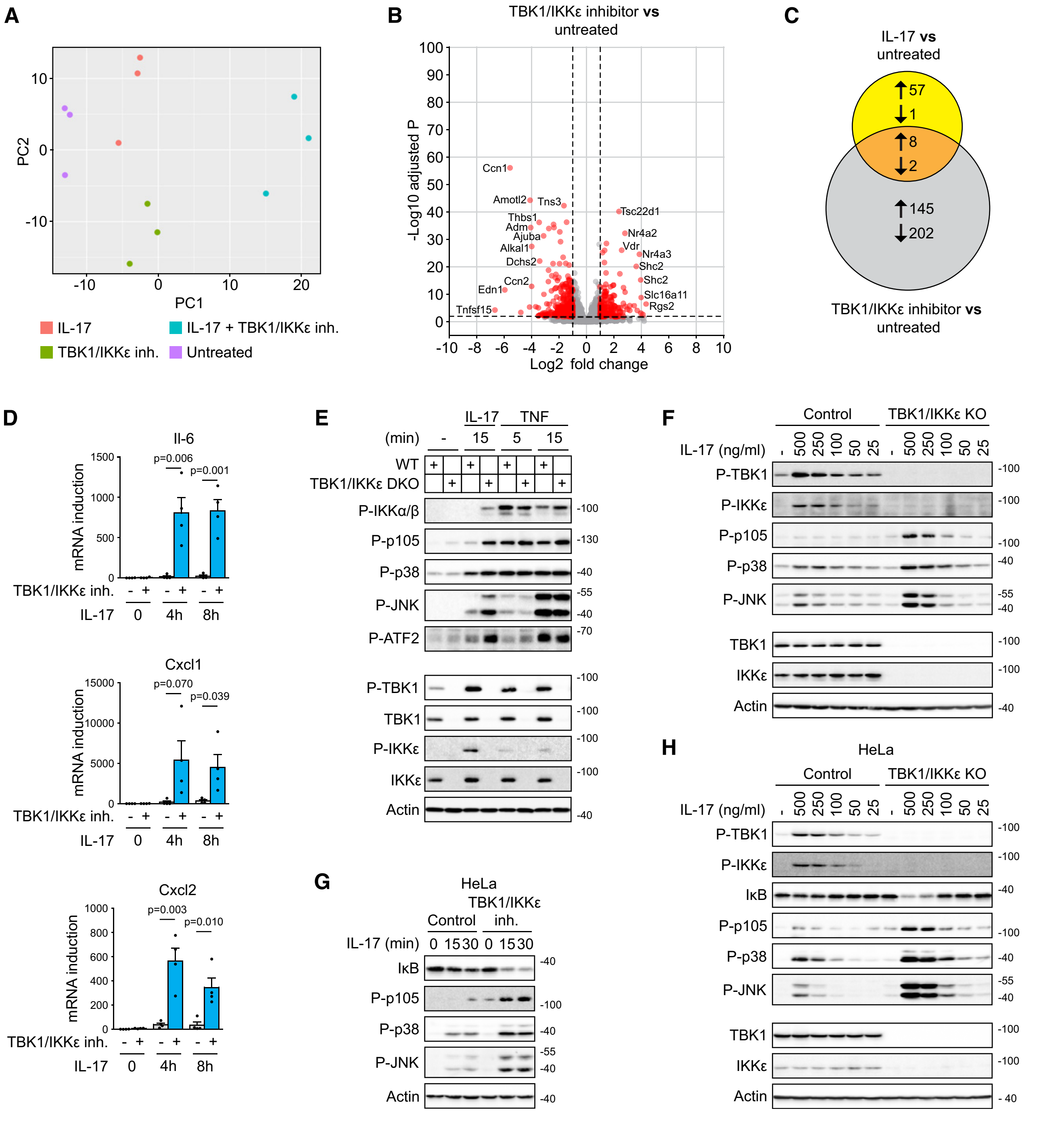

Figure EV2.

© 2020 The Authors

Figure EV2. TBK1 and IKKε function as major inhibitors of IL-17-induced signaling and transcriptional response.

A ST2 cells were incubated with or without TBK1/IKKε inhibitor MRT67307 (2 μM) for 30 min and subsequently were left untreated or stimulated for 2 h with IL-17 (500 ng/ml). mRNA was isolated and subjected to RNA sequencing. The principal component analysis from three independent experiments is shown.

B Analysis of transcriptional response induced by treatment of ST2 cells with TBK1/IKKε inhibitor only. In red are transcripts considered to be significantly changed ($\log_2$ fold change > 1 or < −1, $-\log_{10}$ Benjamini–Hochberg adjusted *P*-value > 2, based on analysis of three independent experiments). Names of several significantly upregulated transcripts are indicated.

C The Venn diagram representing the number of significantly changed transcripts upon treatment of cells with TBK1/IKKε inhibitor alone or with IL-17 alone.

D ST2 cells pretreated or not with TBK1/IKKε inhibitor MRT67307 (2 μM) were left unstimulated or stimulated with IL-17 (500 ng/ml) for 4 or 8 h, and induction of mRNA for selected genes was analyzed by real-time PCR. Mean + SEM from four independent experiments is shown, and statistical significance was determined using unpaired two-tailed Student's *t*-test.

E ST2 wild types or TBK1/IKKε DKO cells were stimulated with IL-17 (500 ng/ml) or TNF (50 ng/ml) for indicated time points and lysates were analyzed by immunoblotting.

F ST2 wild types or TBK1/IKKε DKO cells were stimulated with indicated concentration of IL-17 for 15 min and lysates were analyzed by immunoblotting.

G HeLa cells pretreated or not with TBK1/IKKε inhibitor MRT67307 (2 μM) were stimulated with IL17 (500 ng/ml) for indicated time points and lysates were analyzed by immunoblotting.

H HeLla wild types or TBK1/IKKε DKO cells were stimulated with indicated concentration of IL-17 for 15 min and lysates were analyzed by immunoblotting.

Data information: Immunoblot results are representative of two (G, H) or three (F) independent experiments.
Source data are available online for this figure.

© 2020 The Authors

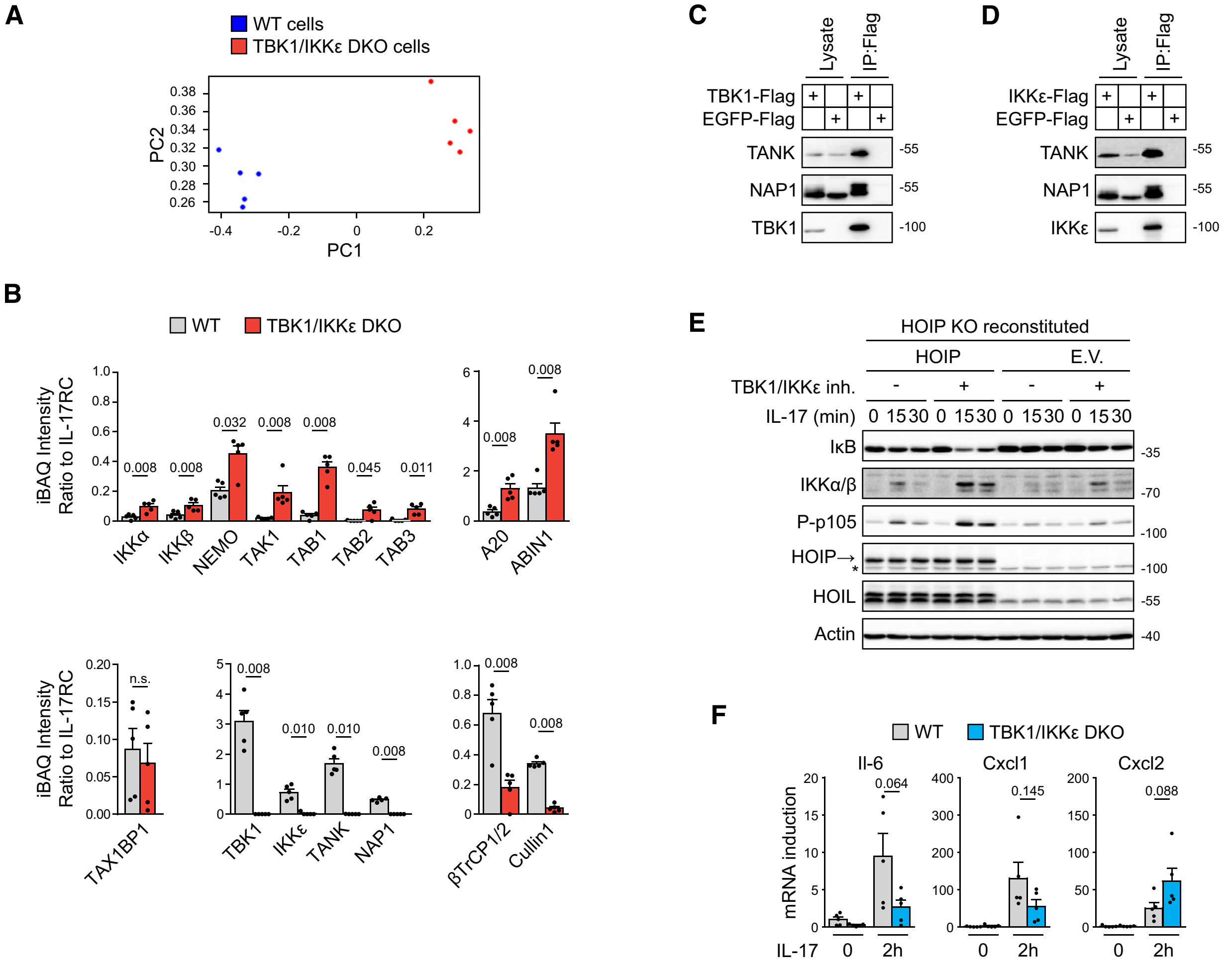

Figure EV3. Ablation of TBK1 and IKKε leads to markedly changed composition of IL-17RSC.

A, B ST2 wild-type or TBK1/IKKε DKO cells were stimulated for 15 min with SF-IL-17 (500 ng/ml), solubilized and IL-17RSC was isolated via consecutive Flag and Strep immunoprecipitation and analyzed by MS. (A) The principal component analysis of five independent experiments. (B) The ratio between iBAQ intensities of selected IL-17RSC components to iBAQ intensity of IL-17RC. Mean + SEM from five independent experiments is shown, and statistical significance was determined by two-tailed Mann-Whitney test.

C, D ST2 TBK1/IKKε DKO cells were reconstituted with Flag-tagged TBK1 (C) IKKε (D) or GFP that serves as negative control. Cell lysates were subjected to anti-Flag immunoprecipitation and analyzed via immunoblotting.

E HOIP KO cells reconstituted with HOIP(WT) or empty vector were pretreated with TBK1/IKKε inhibitor MRT67307 (2 μM), stimulated with IL-17 (500 ng/ml) as indicated and analyzed by immunoblotting. *indicates nonspecific band.

F ST2 wild types or TBK1/IKKε DKO were stimulated with IL-17 (500 ng/ml) for 2 h and induction of mRNA for selected genes was analyzed by real-time PCR. Mean + SEM from five independent experiments is shown, and statistical significance was determined using unpaired two-tailed Student's *t*-test.

Data information: Immunoblot results are representative of two (C, D, E) independent experiments.
Source data are available online for this figure.

© 2020 The Authors

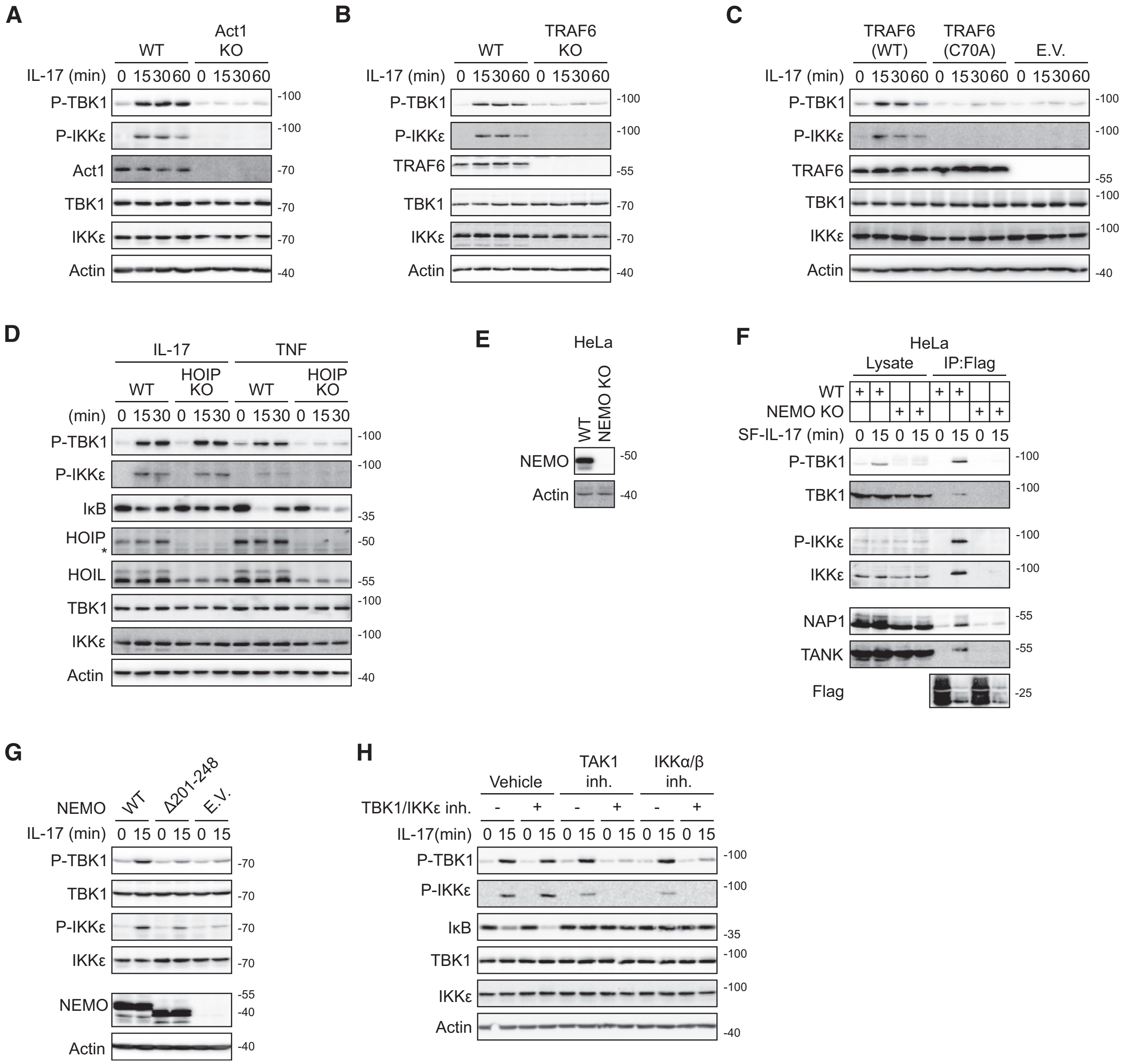

Figure EV4. IL-17-induced TBK1 and IKKε activation requires TRAF6 and NEMO, but not HOIP.

A, B ST2 cells wild-type, ACT1 KO (A), or TRAF6 KO (B) were stimulated for indicated time points with IL-17 (500 ng/ml) and lysates were analyzed by immunoblotting.

C ST2 cells deficient in TRAF6 were reconstituted with TRAF6(WT), enzymatically inactive TRAF6(C70), or empty vector and stimulated with IL-17 (500 ng/ml) for indicated time points. Lysates were analyzed by immunoblotting.

D ST2 cells wild-type or HOIP KO were stimulated with IL-17 (500 ng/ml) or TNF (50 ng/ml) as indicated and lysates were analyzed by immunoblotting. *indicates unspecific band.

E NEMO-deficient HeLa cells were analyzed by immunoblotting.

F HeLa wild-type or NEMO KO cells were stimulated with SF-IL-17 for 15 min or were left unstimulated and IL-17 was added post-lysis. Lysates were subjected to anti-Flag immunoprecipitation to isolate IL-17RSC, and samples were analyzed by immunoblotting.

G NEMO-deficient ST2 cells were reconstituted with NEMO(WT), NEMO (Δ201–248) or empty vector and stimulated with IL-17 (500 ng/ml) as indicated. Activation of signaling pathways was analyzed upon cell lysis by immunoblotting.

H ST2 cells were pretreated with either IKKα/IKKβ inhibitor TPCA1 (10 μM), TAK1 inhibitor 7-oxozeanol (2 μM), TBK1/IKKε inhibitor MRT67307 (2 μM), or their combination as indicated, stimulated or not with IL-17 (500 ng/ml) for 15 min and analyzed by immunoblotting.

Data information: Experiments are representative of two (A, C, D, H) or three (B, F, G) independent experiments.
Source data are available online for this figure.

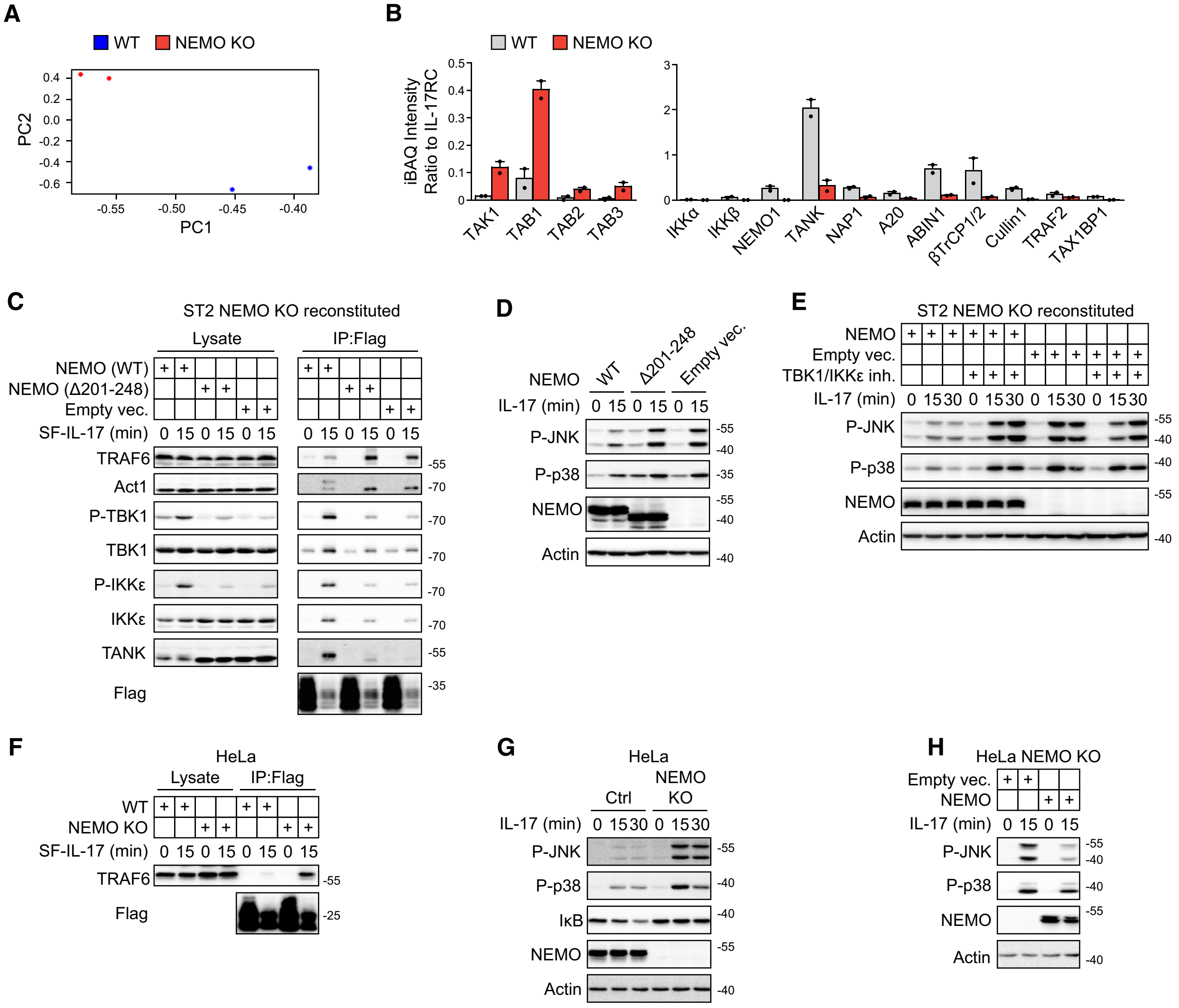

Figure EV5. NEMO mediates recruitment of TBK1 and IKKε to inhibit IL-17RSC complex assembly and downstream signaling.

A, B ST2 wild-type or NEMO KO cells were stimulated for 15 min with SF-IL-17 (500 ng/ml), solubilized and IL-17RSC was isolated via consecutive Flag and Strep immunoprecipitation and analyzed by MS. (A) The principal component analysis of two independent experiments. (B) The ratio between iBAQ intensities of selected IL-17RSC components to iBAQ intensity of IL-17RC. Mean + SEM from two independent experiments is shown.

C, D NEMO-deficient ST2 cells were reconstituted with NEMO(WT), NEMO (Δ201–248) lacking TANK/NAP1-binding site or empty vector and stimulated with SF-IL-17 (500 ng/ml) for 15 min or were left unstimulated and IL-17 was added post-lysis. Lysates were subjected to anti-Flag immunoprecipitation to isolate IL-17RSC (C) or tested for the activation of signaling pathways (D) and samples were analyzed by immunoblotting.

E NEMO-deficient ST2 cells were reconstituted with NEMO(WT) or empty vector, treated or not with TBK1/IKKε inhibitor MRT67307 (2 μM) and stimulated with SF-IL-17 (500 ng/ml) as indicated. Activation of signaling pathways was analyzed upon cell lysis by immunoblotting.

F, G HeLa wild-type or NEMO KO cells were stimulated with SF-IL-17 (500 ng/ml) for 15 min or were left unstimulated and IL-17 was added post-lysis. Lysates were subjected to anti-Flag immunoprecipitation to isolate IL-17RSC (F) or tested for the activation of signaling pathways (G) and samples were analyzed by immunoblotting.

H NEMO-deficient HeLa cells were reconstituted with NEMO(WT) or empty vector and stimulated with IL-17 (500 ng/ml) as indicated. Cells were solubilized and analyzed by immunoblotting.

Data information: Immunoblot results are representative of two (C, D, E, G, H) or four (F) independent experiments.
Source data are available online for this figure.

 © 2020 The Authors

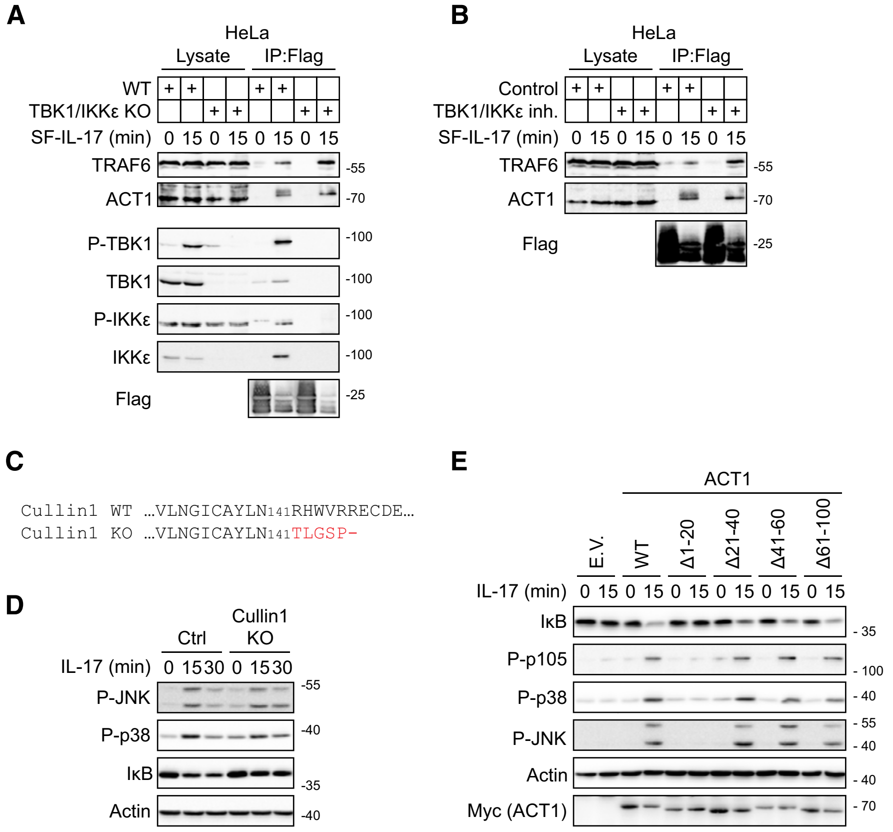

Figure EV6. Activity of TBK1 and IKKε kinases promotes release of TRAF6 from the IL-17RSC.

A HeLa wild-type or TBK1/IKKε DKO cells were stimulated with SF-IL-17 as indicated, solubilized, and subjected to anti-Flag immunoprecipitation to isolate IL-17RSC. Samples were analyzed by immunoblotting.

B HeLa cells were treated or not with TBK1/IKKε inhibitor MRT67307 (2 μM) and stimulated with SF-IL-17 (500 ng/ml) as indicated. IL-17RSC was isolated by anti-Flag immunoprecipitation and analyzed by immunoblotting.

C Cullin1 KO ST2 cells were prepared by CRISPR/Cas9 and verified by sequencing. Biallelic deletion of 2 base pairs leads to frameshift in Cullin1 coding sequence and premature STOP codon.

D Cullin1 KO ST2 cells were stimulated for 15 min with IL-17 (500 ng/ml), and lysates were analyzed by immunoblotting.

E ACT1-deficient ST2 cells were reconstituted with ACT1(WT) or ACT1 harboring indicated deletions or empty vector. Cells were stimulated for 15 min with IL-17 (500 ng/ml), and lysates were analyzed by immunoblotting.

Data information: Data are representative of two (A, B, E) or four (D) independent experiments.
Source data are available online for this figure.

© 2020 The Authors
